# The nuclear receptor RORα preserves cardiomyocyte mitochondrial function by regulating caveolin-3-mediated mitophagy

**DOI:** 10.1016/j.jbc.2021.101358

**Published:** 2021-10-28

**Authors:** Ju Youn Beak, Hong Soon Kang, Wei Huang, Rishi Deshmukh, Seok Jae Hong, Nishi Kadakia, Amir Aghajanian, Kevin Gerrish, Anton Jetten, Brian Jensen

**Affiliations:** 1 McAllister Heart Institute, University of North Carolina School of Medicine, Chapel Hill, North Carolina, USA; 2 Immunity, Inflammation and Disease Laboratory, National Institute of Environmental Health Sciences, National Institutes of Health, Durham, North Carolina, USA; 3 Campbell University School of Osteopathic Medicine, Lillington, North Carolina, USA; 4 Department of Cell Biology and Physiology, University of North Carolina School of Medicine, Chapel Hill, North Carolina, USA; 5 Molecular Genomics Core Laboratory, National Institute of Environmental Health Sciences, Durham, North Carolina, USA; 6 Division of Cardiology, University of North Carolina School of Medicine, Chapel Hill, North Carolina, USA

**Keywords:** mitophagy, RAR-related orphan receptor alpha (ROR-alpha), cardiomyocyte mitochondrial physiology, caveolin-3, hypoxia, Ang II, angiotensin II, BFA1, bafilomycin A1, Bnip3, BCL2 interacting protein 3, Cav-3, caveolin-3, ChIP, chromatin immunoprecipitation, CMKO, cardiomyocyte-specific RORα KO, HIF-1α, hypoxia-inducible factor-1α, HRP, horseradish peroxidase, Hsp60, heat shock protein 60, JC-1, 5, 5′, 6, 6′-tetrachloro-1, 1′, 3, 3′-tetraethylbenzimidazolylcarbocyanine iodide, LC3B-II, microtubule-associated protein light chain beta 3-II, LDH, lactate dehydrogenase, mPTP, mitochondrial permeability transition pore, mt-Keima, mitochondrial-targeted Keima, NRVMs, neonatal rat ventricular myocytes, OCR, oxygen consumption rate, PFA, paraformaldehyde, PINK1, PTEN-induced putative kinase 1, qRT-PCR, quantitative RT-PCR, ROR, retinoic acid–related orphan nuclear receptor, ROS, reactive oxygen species, SDH, succinate dehydrogenase, shCtrl, scrambled shRNA, shRORα, shRNA against RORα, TEM, transmission electron microscopy

## Abstract

Preserving optimal mitochondrial function is critical in the heart, which is the most ATP-avid organ in the body. Recently, we showed that global deficiency of the nuclear receptor RORα in the “staggerer” mouse exacerbates angiotensin II–induced cardiac hypertrophy and compromises cardiomyocyte mitochondrial function. However, the mechanisms underlying these observations have not been defined previously. Here, we used pharmacological and genetic gain- and loss-of-function tools to demonstrate that RORα regulates cardiomyocyte mitophagy to preserve mitochondrial abundance and function. We found that cardiomyocyte mitochondria in staggerer mice with lack of functional RORα were less numerous and exhibited fewer mitophagy events than those in WT controls. The hearts of our novel cardiomyocyte-specific RORα KO mouse line demonstrated impaired contractile function, enhanced oxidative stress, increased apoptosis, and reduced autophagic flux relative to Cre(-) littermates. We found that cardiomyocyte mitochondria in “staggerer” mice with lack of functional RORα were upregulated by hypoxia, a classical inducer of mitophagy. The loss of RORα blunted mitophagy and broadly compromised mitochondrial function in normoxic and hypoxic conditions *in vivo* and *in vitro*. We also show that RORα is a direct transcriptional regulator of the mitophagy mediator caveolin-3 in cardiomyocytes and that enhanced expression of RORα increases caveolin-3 abundance and enhances mitophagy. Finally, knockdown of RORα impairs cardiomyocyte mitophagy, compromises mitochondrial function, and induces apoptosis, but these defects could be rescued by caveolin-3 overexpression. Collectively, these findings reveal a novel role for RORα in regulating mitophagy through caveolin-3 and expand our currently limited understanding of the mechanisms underlying RORα-mediated cardioprotection.

The heart consumes more ATP than any other organ by virtue of its requirement for constant cardiomyocyte contraction and relaxation. As such, maintenance of a large and optimally functional pool of mitochondria is essential to normal cardiac physiology. Compromised mitochondrial function leads to energy deprivation and enhanced oxidative stress, both of which are central to the pathobiology of heart failure (1). The cardiac mitochondrial pool is preserved through careful orchestration of mitochondrial biogenesis and the clearance of dysfunctional mitochondria, chiefly through selective mitochondrial autophagy, or mitophagy (2). Nuclear receptors increasingly are recognized as central transcriptional regulators of these critical processes in the heart (3).

The retinoic acid–related orphan nuclear receptor (ROR) subfamily consists of three members, RORα, RORβ, and RORγ (*NR1F1-3*), with highly tissue-specific and context-dependent expression. RORα plays well-described roles in regulating circadian rhythm and T cell development (4). RORα is also known to regulate multiple aspects of mitochondrial function and metabolism including glucose metabolism in hepatoma cells (5) and lipid homeostasis in the liver (6) and in macrophages (7). The RORα agonist nobiletin enhances transcription of mitochondrial respiratory chain complexes leading to increased metabolic fitness in skeletal muscle (8). However, there was no recognized function for RORα in the heart before two recent reports from Pu *et al.*, who showed that RORα expression is protective in myocardial ischemia/reperfusion and diabetic cardiomyopathy (9, 10). We extended those findings in the angiotensin II (Ang II)-induced cardiac injury model, demonstrating that “staggerer” mice that globally lack functional RORα (RORα^sg/sg^) develop exaggerated myocardial hypertrophy and contractile dysfunction after Ang II exposure (11). We also made the novel observation that RORα deficiency was associated with decreased mitochondrial abundance, ATP depletion, and enhanced oxidative stress in cardiomyocytes. The mechanisms underlying these findings and putative functions for RORα in uninjured cardiomyocytes remain unclear.

In this study, we use *in vivo* and *in vitro* approaches, including a new cardiomyocyte-specific RORα KO (CMKO) mouse, to identify a novel role for RORα in regulating cardiomyocyte mitophagy. We use hypoxia as an *in vivo* and *in vitro* stressor to test the role of RORα in the cardiac response to stress because hypoxia is known to stimulate mitophagy and because the cellular response to hypoxia is critical to clinically important cardiac conditions such as ischemia-reperfusion injury and myocardial infarction. The loss of cardiomyocyte RORα is associated with abnormal mitochondrial structure and physiology, and RORα CMKO hearts exhibit numerous indicators of mitochondrial dysfunction. Mechanistically, we show that these abnormalities arise at least in part from defects in mitophagy due to impaired transcriptional regulation of caveolin-3 (Cav-3) in the absence of RORα. These new findings may account for our previous observation of energy deprivation in the hearts of RORα^sg/sg^ mice and emphasize the emerging importance of RORα as a cardioprotective nuclear receptor.

## Results

### Mitochondria in RORα^sg/sg^ mouse hearts are less abundant and structurally abnormal with fewer mitophagic events

**Figure 1:**
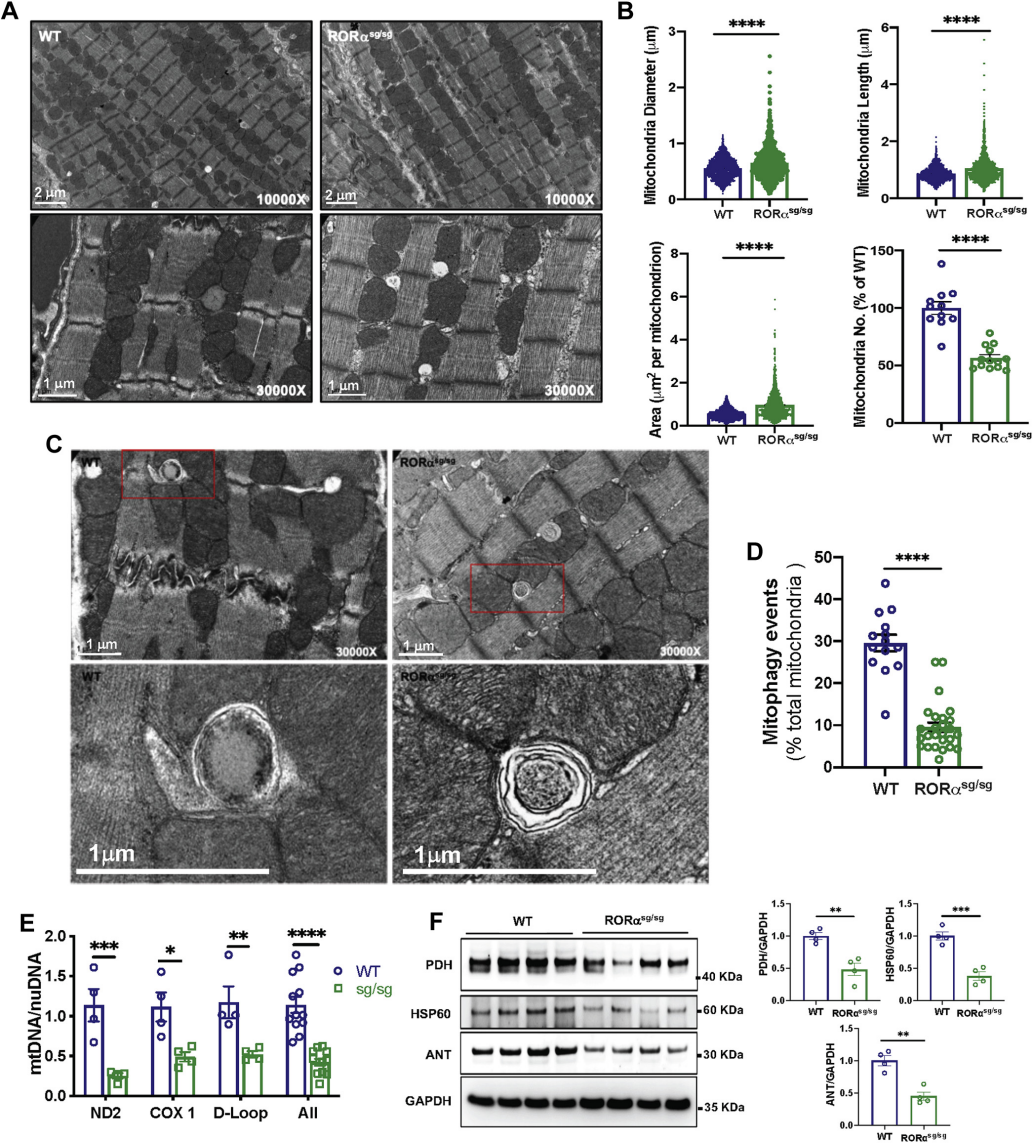
**Mitochondrial abundance is lower in RORα**^**sg/sg**^**than WT mouse hearts.***A*, transmission electron microscopy (TEM) of WT and RORα^sg/sg^ hearts; *B*, quantitative analysis of TEM mitochondrial abundance and size using ImageJ with the number of mitochondria analyzed (n = 2 hearts from 4-month-old mice per group). *C*, representative TEM image of mitochondria undergoing lysosomal degradation in WT and RORα^sg/sg^ hearts. *D*, quantitation of mitochondria undergoing lysosomal degradation using ImageJ. *E*, quantitative PCR for mitochondrial DNA (mtDNA) normalized to nuclear DNA (nuDNA). *F*, immunoblot of mitochondrial proteins pyruvate dehydrogenase (PDH), heat shock protein 60 (HSP60), and adenine nucleotide transferase (ANT) normalized to the cytoplasmic control GAPDH with summary densitometry using ImageJ. ∗*p* < 0.05, ∗∗*p* < 0.01, ∗∗∗*p* < 0.001, and ∗∗∗∗*p* < 0.0001 by unpaired *t* test (*B*, *D*, and *F*) or one-way ANOVA (*E*).

Our recent study demonstrated that RORα protects cardiomyocytes against Ang II-induced hypertrophy by maintaining the mitochondrial number and function (11). To explore the potential roles for RORα in preserving the cardiomyocyte mitochondrial pool, we examined WT and RORα^sg/sg^ hearts from 4-month-old mice using transmission electron microscopy (TEM). We found that the subsarcolemmal mitochondria in RORα^sg/sg^ left ventricular myocardium were 44% less abundant but 43% larger than in the left ventricular myocardium from WT littermates (Fig. 1, *A* and *B*), broadly suggestive of defects in mitochondrial quality control. We also analyzed the TEM images for mitochondria engulfed in double-membrane lysosomes, a finding that is indicative of active autophagy, and found 68% fewer of these structures in the RORα^sg/sg^ than the WT hearts. (Fig. 1, *C* and *D*). Quantitative PCR for mitochondrial DNA genes (ND2, Cox1, D-loop, Fig. 1*E*) and immunoblotting for proteins with mitochondrially restricted expression (pyruvate dehydrogenase, heat shock protein 60 [Hsp60], adenine nucleotide transferase, Fig. 1*F*) corroborated our TEM findings that RORα^sg/sg^ hearts contained fewer mitochondria than WT hearts. Taken together, these findings suggest that defects in mitophagy may contribute to mitochondrial depletion and dysfunction in the absence of RORα.

### RORα regulates expression of mitophagy mediators in normoxic and hypoxic conditions *in vivo* and *in vitro*

To explore at a transcriptional level whether alterations in mitochondrial biogenesis, dynamics, or mitophagy might determine the compromised functional and morphological mitochondrial phenotype in the RORα^sg/sg^ mouse hearts, we performed quantitative RT-PCR (qRT-PCR) for selected genes critical to each process. The expression of *PGC1α*, *Gabpa*, and *TFAM* (mitochondrial biogenesis, Fig. S1*A*) was somewhat reduced, as was the expression of *Opa1* (mitochondrial dynamics, Fig. S1*B*). More profound transcriptional differences were found in the autophagy-related genes autophagy and beclin 1 regulator 1 (*Ambra1*), autophagy-related 7 (*Atg7*), *Atg10*, and beclin 1 (*Becn1*) (Fig. S1*C*). Using qRT-PCR, we also confirmed decreased expression of multiple key electron transport genes, further corroborating the presence of an RORα-related mitochondrial defect (Fig. S1*D*). These findings coupled with the morphological abnormalities seen on TEM encouraged us to determine whether and how RORα regulates cardiomyocyte mitophagy.

**Figure 2:**
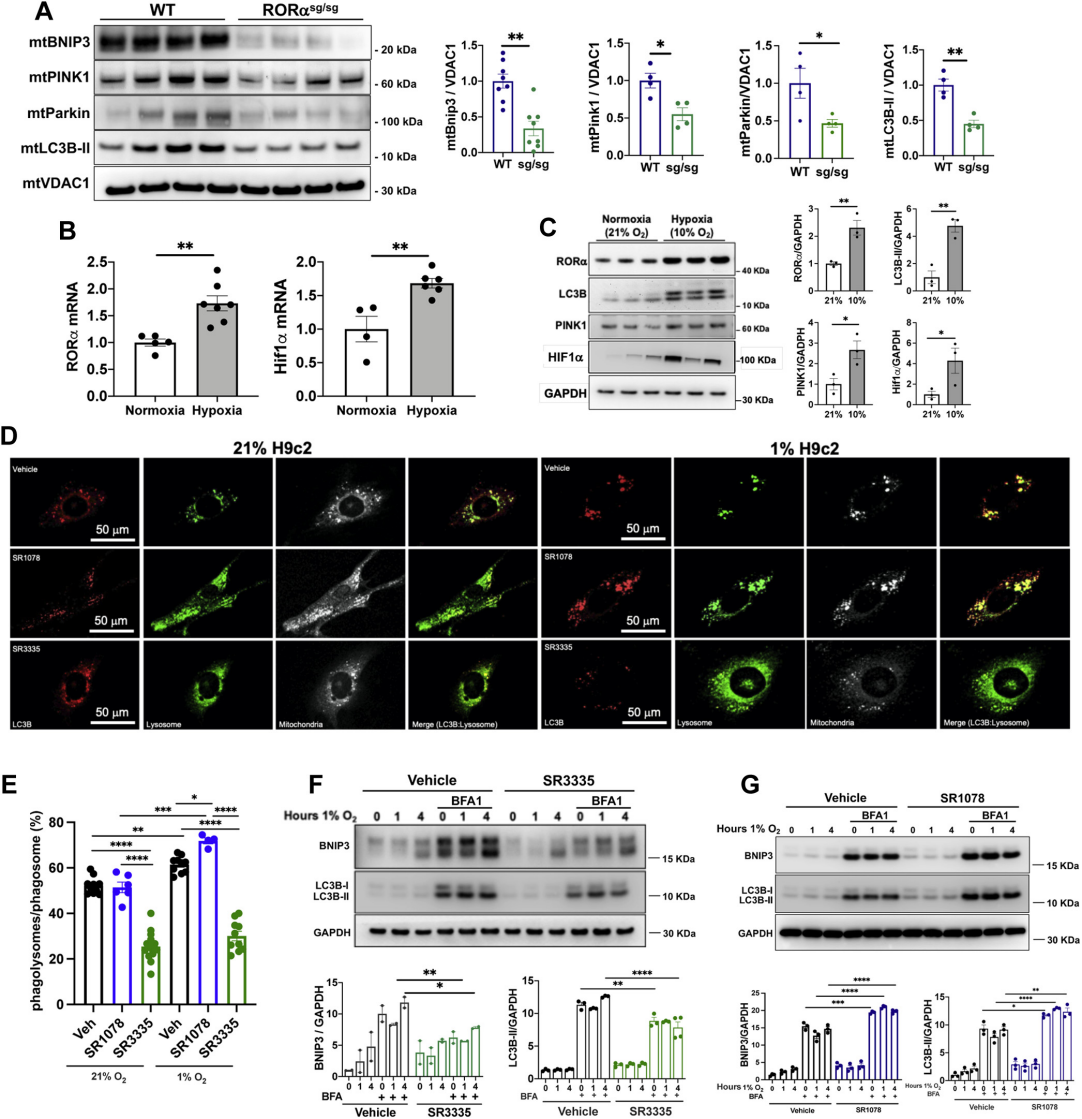
**Hypoxia-induced mitophagy is blunted by the absence of RORα*****in vivo*****and*****in vitro*****.***A*, immunoblotting of mitochondrial fraction from WT and RORα^sg/sg^ heart lysates. *B*, quantitative RT-PCR on hearts of mice exposed to normoxia or hypoxia (10% O_2_) for 24 h, normalized to *Actb* and *Tbp*. *C*, immunoblotting of heart lysates of mice exposed to normoxia or hypoxia. *D*, immunofluorescence staining of normoxic or hypoxic (1% O_2_ for 24 h) H9c2 cardiomyoblasts exposed to vehicle, RORα agonist SR1078 (10 mM), or RORα inverse agonist SR3335 (20 mM). *E*, quantitative analysis of phagolysosomes as identified by costaining LC3B, the lysosomal stain LysoTracker, and mitochondria using ImageJ. *F*, immunoblotting of NRVM lysates exposed to hypoxia in the presence or absence of bafilomycin A with SR3335 (20 μM) or (*G*) SR1078 (10 μM) with summary densitometry (ImageJ). ∗*p* < 0.05, ∗∗*p* < 0.01, ∗∗∗*p* < 0.001, and ∗∗∗∗*p* < 0.0001 by unpaired *t* test (*A*–*C*) or one-way ANOVA with Tukey's post hoc test (*E*–*G*). BFA, bafilomycin A; Bnip3, BCL2 interacting protein 3; Hif1a, hypoxia-inducible factor1 alpha; LC3, microtubule-associated protein 1A/1B light chain 3; Pink1, PTEN-induced kinase 1; VDAC1, voltage-dependent anion channel 1.

Mitophagy is carried out primarily through two well-defined pathways: PTEN-induced putative kinase 1 (PINK1)/Parkin and BCL2 interacting protein 3 (Bnip3)/NIX. Activation of either of these pathways can target mitochondria for autophagy by linking to microtubule-associated protein light chain beta 3-II (LC3B-II) on autophagosome membranes (12). To better understand whether RORα might affect mitophagy, we examined the mitochondrial fraction of WT and RORα^sg/sg^ mouse hearts for these canonical mitophagy mediators by Western blot and found that the absence of functional RORα was associated with markedly decreased abundance of mitochondrial Bnip3, PINK1, and LC3B-II (Fig. 2*A*). Confirmation of the purity of the subcellular fraction lysates and immunoblotting of whole-cell lysates are found in Figure S2, *A* and *B*, respectively.

Oxygen tension is a potent regulator of mitochondrial function in the heart, and protecting mitochondrial function is critical when the myocardium becomes ischemic (13). Under hypoxic conditions, such as myocardial infarction, mitophagy is upregulated to promote clearance of dysfunctional mitochondria. Failure to induce mitophagy can lead to exacerbated cardiomyocyte injury and impaired survival (14). To assess the *in vivo* function of RORα in low oxygen tension, we subjected WT mice to hypoxia (10% O_2_ for 24 h). Hypoxia led to nearly 2-fold upregulation of myocardial RORα mRNA expression, similar in magnitude to the classical hypoxia marker, Hif1α (Fig. 2*B*). Immunoblots of heart lysates revealed that hypoxic upregulation of RORα and Hif1α was coupled with significant increases in LC3B-II and PINK1 expression, consistent with induction of mitophagy (Fig. 2*C*).

Next, we sought to determine whether pharmacological manipulation of RORα activity *in vitro* could recapitulate our genetic loss-of-function findings from our *in vivo* models. We exposed H9c2 rat ventricular myoblasts to hypoxia (1% O_2_) in the presence and absence of the RORα inverse agonist SR3335 or agonist SR1078 and then quantified the number of phagolysosomes costained for LC3B (red) and the lysosomal stain LysoTracker (green) (Fig. 2*D*). The RORα agonist SR1078 increased, and the RORα inverse agonist SR3335 decreased phagolysosome events in both normoxia and hypoxia (Fig. 2*E*), consistent with our *in vivo* findings. We then exposed normoxic and hypoxic neonatal rat ventricular myocytes (NRVMs) treated with SR3335 or SR1078 to bafilomycin A1 (BFA1), which interferes with autolysosome acidification and autophagosome formation through inhibition of V-ATPase (15), allowing measurement of autophagic flux as indicated by the abundance of LC3B-II (16). As expected, bafilomycin A increased the expression of the mitophagy adaptor BNIP3, as well as LC3B-II, in vehicle-treated NRVMs. The RORα inverse agonist SR3335 abrogated upregulation of BNIP3 and blunted the LC3B-II increase (Fig. 2*F*), whereas the agonist SR1078 increased LC3B-II and BNIP3 expression (Fig. 2*G*), again suggesting that RORα regulates hypoxic autophagic flux.

Collectively, these findings indicate that RORα expression facilitates cytoprotective mitophagy in normoxia and hypoxia, corroborating our morphological (Fig. 1) findings.

### Cardiomyocyte mitophagy is compromised *in vivo* in a novel CMKO mouse

**Figure 3:**
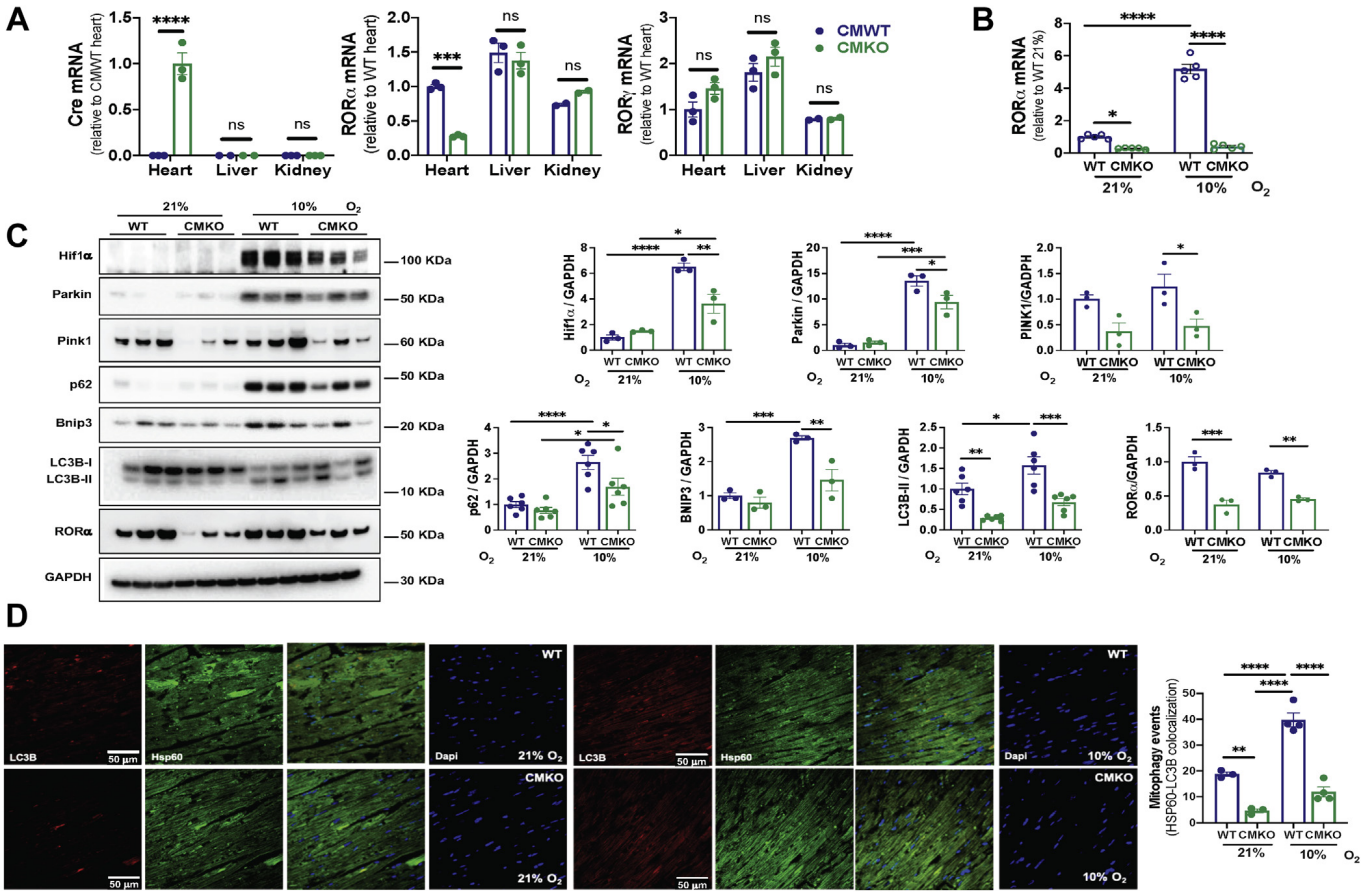
**Mitophagy is limited in cardiomyocyte-specific RORα KO mouse hearts.***A*, quantitative RT-PCR (qRT-PCR) for Cre, RORα, and RORγ genes in the heart, liver, and kidney from CMWT and cardiomyocyte-specific RORα KO mice (n = 3 per group). *B*, qRT-PCR for RORα in the heart tissue from CMWT and CMKO mice exposed to normoxia (21% O_2_) or to hypoxia (10% O_2_) for 8 h. *C*, immunoblotting from WT and CMKO heart lysates with summary densitometry (ImageJ). *D*, representative LC3B immunofluorescence images of mouse heart sections (≥3 per mouse) and ImageJ summary quantification. ∗*p* < 0.05, ∗∗*p* < 0.01, ∗∗∗*p* < 0.001, and ∗∗∗∗*p* < 0.0001 by unpaired *t* test (*A*) or one-way ANOVA with Tukey's post hoc test (*B*–*D*). Atg7, autophagy related 7; Atg10, autophagy related 10; Bnip3, BCL2 interacting protein 3; Cre, Cre recombinase; DAPI, 4′,6-diamidino-2-phenylindole; Hif1a, hypoxia inducible factor1 alpha; Hsp60, heat shock protein 60; LC3B, microtubule-associated protein 1A/1B light chain 3; PINK1, PTEN-induced kinase 1; p62, SQSTM1.

The RORα^sg/sg^ “staggerer” mouse is a well-validated genetic loss-of-function model, but it exhibits numerous extracardiac phenotypes that could potentially influence our study of mitophagy in the heart. Although our *in vitro* studies support cardiomyocyte autonomous functions for RORα, we created a CMKO mouse, breeding floxed RORα mice with mice expressing Cre-recombinase under the control of the alpha myosin heavy-chain promoter, to extend those findings *in vivo* and eliminate potential contributions from extracardiac phenotypes. Cre mRNA was detected only in the heart, and RORα mRNA abundance was 80% lower in the CMKO than CMWT (*Cre*^*−*^*flox*^*+/+*^) hearts. Expression in the liver and kidney was unaffected. Collectively, these data validated our breeding strategy. We found no compensatory changes in RORγ transcriptional expression (Fig. 3*A*). Hypoxia increased RORα transcripts in WT but not in CMKO mouse hearts (Fig. 3*B*). Immunoblotting revealed substantially lower levels of RORα in CMKO than WT hearts (Fig. 3*C*). Residual expression of RORα in CMKO hearts likely is attributable to the known expression of RORα in cardiac fibroblasts (11), smooth muscle cells (17, 18), and endothelial cells (18).

We then examined the effect of cardiomyocyte-specific RORα deletion on the *in vivo* expression of several central mediators of mitophagy induction and maintenance. RNA was isolated from hearts of CMWT and CMKO mice that were housed in normoxic (21% O_2_) or hypoxic (10% O_2_) conditions for 8 h. Abundance of the transcripts encoding Atg10 and Atg7, autophagy receptor p62, and PINK1 was higher in hypoxic than normoxic CMWT hearts, as predicted. However, the hypoxia-induced increase in these critical autophagy mediators was abrogated in CMKO mouse hearts (Fig. S3*A*), consistent with the concept that RORα contributes to hypoxic transcriptional upregulation of autophagy mediators. qRT-PCR detected no significant differences in classic regulators of mitochondrial biogenesis between CMWT and CMKO mouse hearts (Fig. S3*B*). Immunoblotting demonstrated the expected increases in Parkin, p62, Bnip3, and LC3B-II in hypoxic CMWT hearts, but these changes were blunted in CMKO hearts (Fig. 3*C*).

We next sought to examine the consequences of these molecular differences on mitophagy. In both normoxic and hypoxic conditions, CMKO hearts exhibited 75% fewer mitophagy events than CMWT hearts as defined by costaining for LC3B and the mitochondria marker Hsp60 (Fig. 3*D*). Summary analysis of these data indicated that hypoxia induced a roughly 2-fold increase in mitophagy in both CMWT and CMKO hearts, suggesting that hypoxia induces mitophagy through both RORα-dependent and RORα-independent pathways. Mitophagosomes were 70% less abundant in CMKO than CMWT, indicating that RORα is an important regulator of hypoxic mitophagy and that RORα-independent pathways are insufficient to compensate for the absence of RORα.

**Figure 4:**
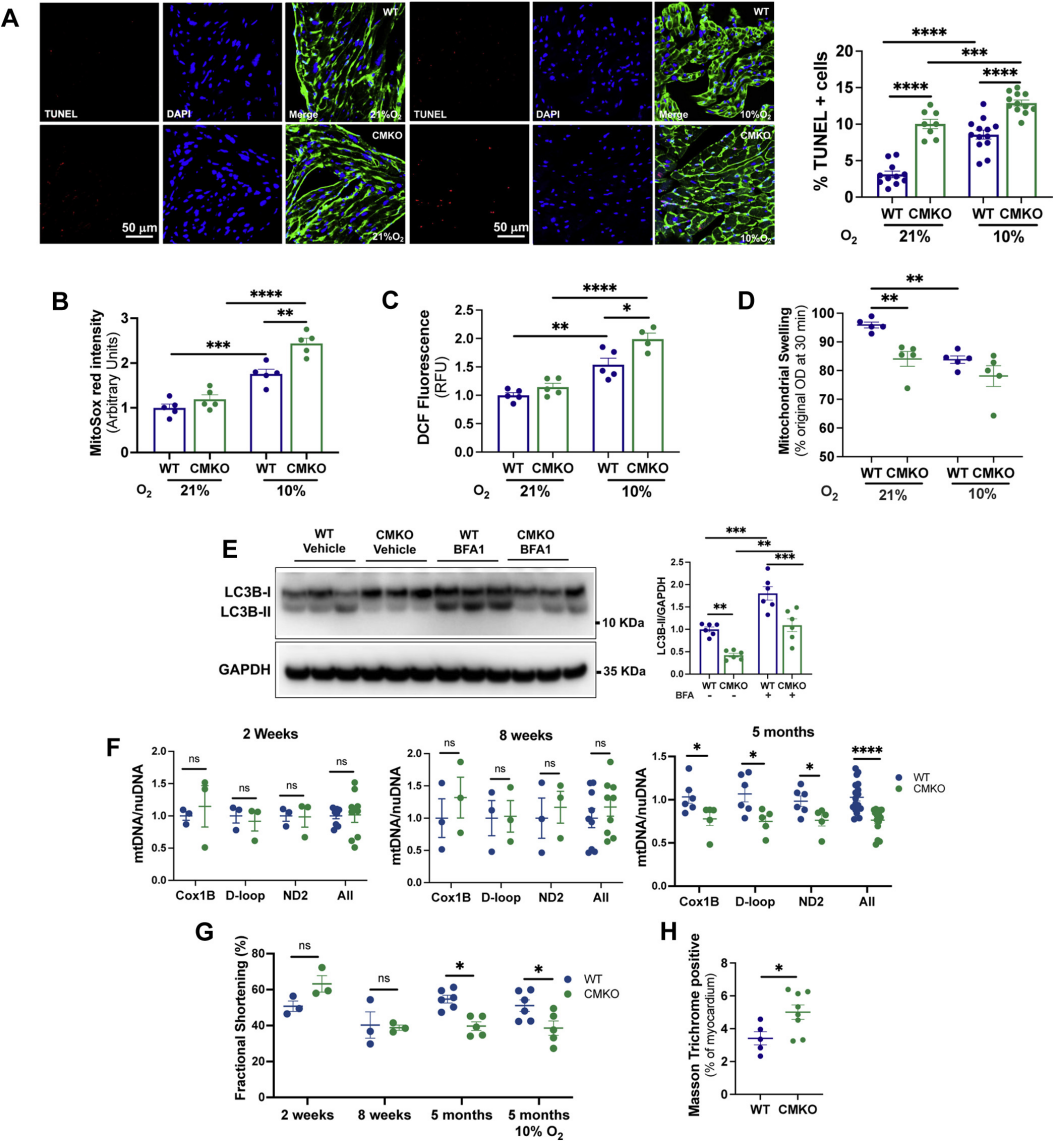
**Cardiomyocyte-specific RORα KO mouse hearts have compromised mitochondrial function and abundance*****in vivo.**A*, TUNEL (*red*), DAPI (*blue*), and wheat germ agglutinin (*green*) staining of WT and CMKO hearts with summary TUNEL+ cells (%); oxidative stress as assayed by (*B*) MitoSOX and (*C*) DCF. *D*, swelling of isolated mouse heart mitochondria in calcium uptake assay. *E*, *in vivo* autophagic flux as measured by immunoblotting for LC3B 2 h after injection of BFA or vehicle. *F*, quantitative PCR for mitochondrial DNA (Cox1B, D-loop, and ND2) relative to nuclear DNA (Actb) by mouse age. *G*, fractional shortening as measured by echocardiography (n = 3–6 per group). *H*, fibrosis as assayed by Masson Trichrome staining. ∗*p* < 0.05, ∗∗*p* < 0.01, and ∗∗∗*p* < 0.001 by one-way ANOVA with Tukey's post hoc test (*A*–*E*) or unpaired *t* test (*F*–*H*). DCF, dichlorofluorescein; LC3B, microtubule-associated protein 1A/1B-light chain 3; ns, not significant; OD, optical density.

We then sought to characterize more fully the physiological effects of this mitophagy defect in CMKO mice. TUNEL staining comparing CMKO and CMWT hearts revealed 3-fold more apoptotic CMKO cells in normoxia and 1.5-fold more apoptotic CMKO cells in hypoxia (*p* < 0.001, Fig. 4*A*). Counterstaining with wheat germ agglutinin to demonstrate cardiomyocyte cell membranes revealed that both cardiomyocytes and nonmyocytes (particularly endothelial cells) exhibited more apoptosis in CMKO than CMWT hearts. Oxidative stress, as assayed by MitoSOX (Fig. 4*B*) and dichlorofluorescein fluorescence (Fig. 4*C*), was more pronounced in hypoxic CMKO than WT hearts, potentially contributing to endothelial cell apoptosis through paracrine effects (19, 20). To extend our findings, we carried out a mitochondrial swelling assay, wherein calcium was added to isolated mitochondria from WT and CMKO hearts to induce opening of the mitochondrial permeability transition pore (mPTP). Mitochondrial swelling, as measured by the change in optical density 30 min after calcium addition, was substantially increased in CMKO compared with WT mitochondria (*p* < 0.01, Fig. 4*D*). These findings suggest the accumulation of defective mitochondria in CMKO hearts, consistent with a defect in mitophagy.

To measure autophagic flux *in vivo*, we sacrificed WT and CMKO mice 2 h after administration of BFA1 *via* intraperitoneal injection. LC3B-II abundance was two-fold higher in WT than in CMKO mouse hearts (Fig. 4*E*), suggesting marked defects in CMKO autophagic flux. Quantitative PCR of 3 mitochondrial DNA genes (mtDNA) normalized to a nuclear DNA control demonstrated that mtDNA (an index of total mitochondrial mass) was less abundant in CMKO than CMWT mice after they reached maturity (Fig. 4*F*).

We then used conscious echocardiography to determine whether the cardiomyocyte-specific loss of RORα conferred effects on contractile function. We found that fractional shortening was 54.7 ± 2.2% in WT but 37.2 ± 3.2% (*p* < 0.01) in 5-month-old CMKO mice maintained continuously in normoxia. Fractional shortening also was lower in hypoxic CMKO than WT mice (*p* < 0.05), although hypoxia itself did not significantly impact contractile function, consistent with previous publications (21). These differences in contractile function only developed after the CMKO mice achieved maturity (Fig. 4*G*). To determine whether the abnormalities in mitochondrial number and function were associated with adverse ventricular remodeling, we used Masson's Trichrome staining (Fig. 4*H*) to find that fibrosis was mildly increased in CMKO hearts (5% myocardial area) compared with CMWT hearts (3% myocardial area).

Collectively, these results substantiate our discovery that RORα regulates mitophagy in the heart. They further indicate that the deficits in mitophagy exhibited by the global RORα^sg/sg^ mouse most likely arise from cardiomyocyte autonomous effects of RORα and that the loss of RORα function in cardiomyocytes has significant adverse effects on cardiac physiology.

### Loss of RORα compromises hypoxic mitophagy and mitochondrial function *in vitro*

**Figure 5:**
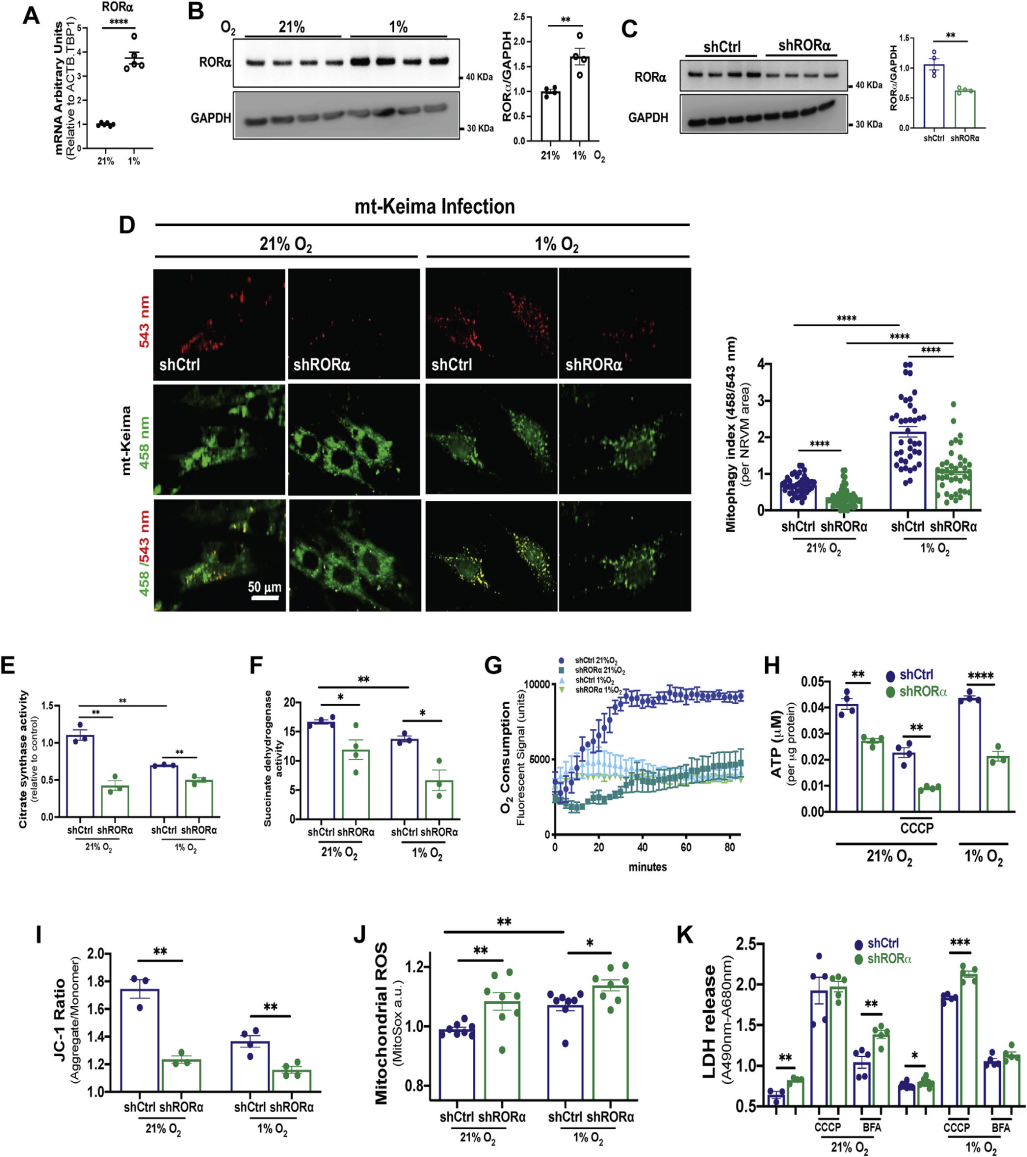
**Knockdown of RORα in cardiomyocytes impairs mitophagy and mitochondrial function*****in vitro*****.***A*, quantitative RT-PCR and (*B*) immunoblotting with summary densitometry for RORα in NRVMs cultured in normoxia and hypoxia; *C*, immunoblotting for RORα in NRVMs infected with lentivirus containing scrambled shRNA (shCtrl) or shRNA against RORα (shRORα). *D*, representative immunofluorescence images of NRVMs infected with the mitophagy indicator, mitochondria-specific Keima (mt-Keima) for 1 h with summary fluorescence quantification using ImageJ. *E*, citrate synthase and (*F*) succinate dehydrogenase activity in NRVMs infected shCtrl or shRORα and then exposed to normoxia (21%) and hypoxia (1%) for 24 h. *G*, oxygen consumption rate in NRVMs infected with lentivirus containing shCtrl or shRORα and then exposed to normoxia or hypoxia. *H*, ATP concentration in NRVMs infected with shCtrl or shRORα in the presence or absence of carbonyl cyanide m-chlorophenyl hydrazine (CCCP), a potent uncoupler of mitochondrial oxidative phosphorylation. *I*, mitochondrial membrane potential assayed using JC-1 (5, 5′, 6, 6′-tetrachloro-1, 1′, 3, 3′-tetraethylbenzimidazolylcarbocyanine iodide) with summary fluorescence using ImageJ. *J*, mitochondrial reactive oxygen species (ROS) assayed using MitoSOX with summary fluorescence using ImageJ. *K*, cell death assayed by lactate dehydrogenase (LDH) release in NRVMs exposed to normoxia or hypoxia in the presence or absence of bafilomycin A (BFA) or CCCP. ∗*p* < 0.05, ∗∗*p* < 0.01, ∗∗∗*p* < 0.001, and ∗∗∗∗*p* < 0.0001 by unpaired *t* test (*A*–*C*) or one-way ANOVA with Tukey's post hoc test (*D*–*K*). NRVMs, neonatal rat ventricular myocytes.

The previous findings encouraged us to further analyze the effect of RORα on mitophagy and mitochondrial function in hypoxic cardiomyocytes. To clarify the effects of hypoxia on RORα expression in cardiomyocytes, as opposed to the pluricellular tissues from our *in vivo* experiments, we exposed NRVMs to 1% O_2_ for 24 h. qRT-PCR showed a roughly 4-fold induction of RORα mRNA (Fig. 5*A*), and immunoblotting demonstrated a roughly 2-fold increase in RORα protein (Fig. 5*B*).

To measure mitophagy in NRVMs, we used mitochondrial-targeted Keima (mt-Keima), a fluorescent protein pH indicator that fluoresces red (543 nm) when introduced to the acidic environment of the lysosome but green (458 nm) at normal or basic pH (22). We infected NRVMs with validated lentivirus (11) containing either shRNA against RORα (shRORα) or scrambled shRNA (shCtrl) (Fig. 5*C*) and found that the mt-Keima fluorescence ratio (543/458 nm) underwent the expected increase under hypoxic conditions but was decreased in both normoxia and hypoxia by RORα knockdown (Fig. 5*D*).

To expand upon our earlier *in vivo* discovery that RORα^sg/sg^ mouse hearts have decreased ATP abundance (11), we used lentiviral shCtrl or shRORα in NRVMs for an *in vitro* loss-of-function approach. Knockdown of RORα in NRVMs decreased the functional mitochondrial mass, as measured by citrate synthase (Fig. 5*E*) and succinate dehydrogenase (SDH) (Fig. 5*F*) activity. The oxygen consumption rate (OCR) was markedly decreased by shRORα (Fig. 5*G*). These metabolic insults collectively contributed to decreased ATP abundance in the absence of functional RORα (Fig. 5*H*). Knockdown of RORα compromised mitochondrial membrane potential as measured by 5, 5′, 6, 6′-tetrachloro-1, 1′, 3, 3′-tetraethylbenzimidazolylcarbocyanine iodide (JC-1) (Fig. 5*I*) and increased mitochondrial reactive oxygen species (ROS) generation as assayed by MitoSOX (Fig. 5*J*). Cell death, as measured by lactate dehydrogenase (LDH) release, was only modestly increased by shRORα (Fig. 5*K*). These findings represent the most extensive evaluation of the effect of RORα on mitochondrial physiology to date and further indicate that RORα contributes to optimal mitochondrial function in both normoxia and hypoxia through maintenance of mitophagy.

### RORα directly binds the Cav**-**3 promoter to regulate hypoxic mitophagy

Cav-3 enhances autophagy and protects mitochondrial function in hypoxic HL-1 cardiomyoblasts (23) and RORα regulates Cav-3 transcription in skeletal muscles (24). Hence, we hypothesized that RORα promotes mitophagy in the heart by regulating the expression of Cav-3.

**Figure 6:**
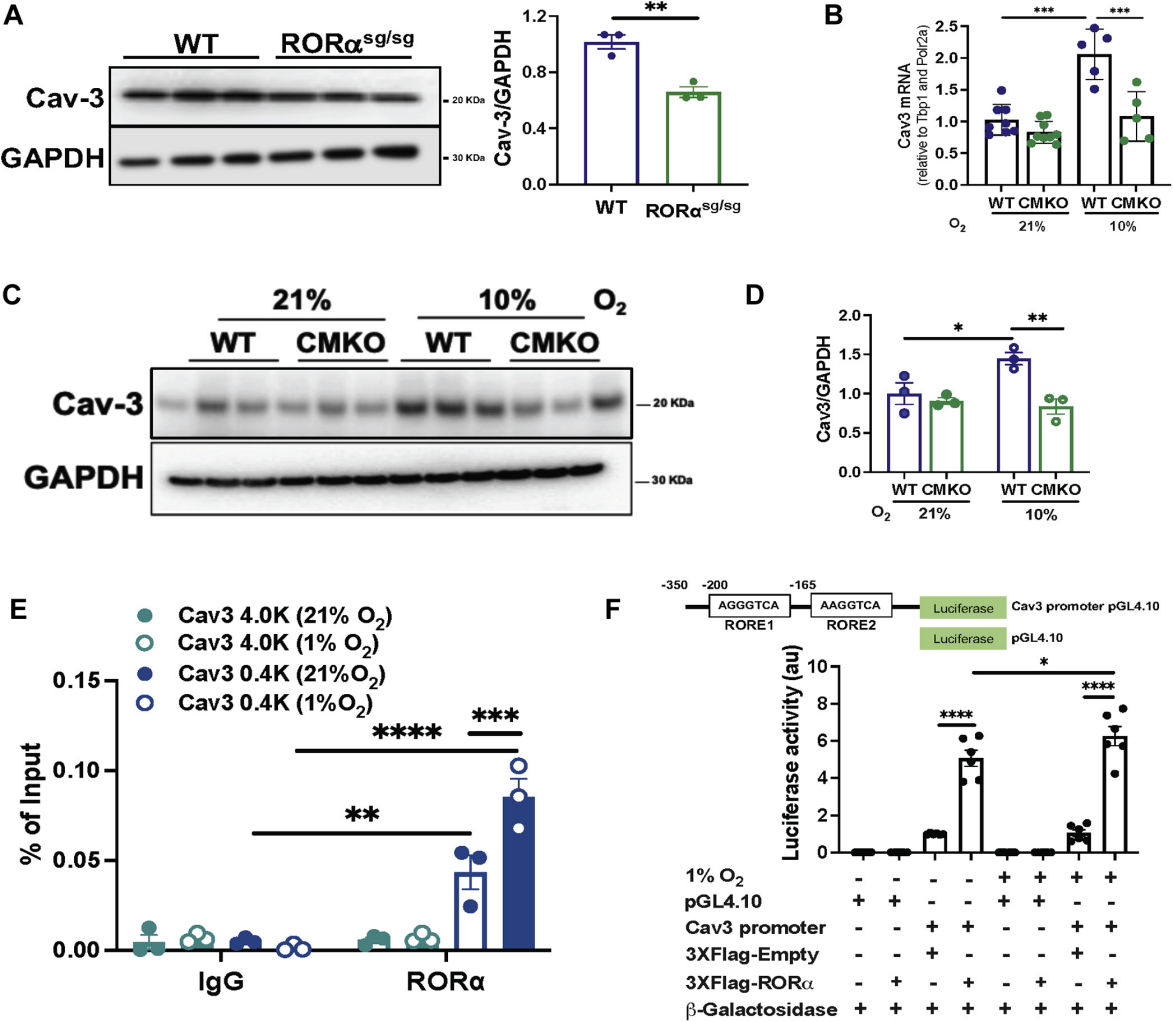
**ROR regulates transcription of caveolin-3.***A*, immunoblotting of heart lysates from WT and sg/sg mouse hearts with summary densitometry (ImageJ). *B*, quantitative RT-PCR for Cav-3 in hearts of WT and CMKO mice exposed to normoxia (21% O_2_ for 8 h) or hypoxia (10% O_2_ for 8 h). *C*, immunoblotting from WT and CMKO heart lysates (n = 3 per group) with (*D*) summary densitometry (ImageJ). *E*, chromatin immunoprecipitation (ChIP)-PCR of Cav-3 in adult mouse ventricular myocytes incubated with anti-RORα or anti-IgG (control) antibodies in normoxic or hypoxic conditions. Two-way ANOVA *p*-value antibody <0.0001, F-value antibody (1,16) = 70.1, *p*-value of the Cav-3 promoter site <0.0001, F-value of the Cav-3 promoter site (3,16) = 23.65, F-value Interaction (3,16) = 28.8, *p*-value interaction <0.0001. *F*, reporter assay measuring luciferase activity in a plasmid containing the Cav-3 promoter in the presence or absence of an RORα-containing plasmid under normoxic or hypoxic conditions. Two-way ANOVA *p*-value RORα <0.0001, F-value (1,20) = 214.2, *p*-value oxygen tension = 0.045, F-value oxygen tension (1,20) = 4.565, *p*-value interaction = 0.060, F-value interaction (1,20) = 3.969. ∗*p* < 0.05, ∗∗*p* < 0.01, ∗∗∗*p* < 0.001, and ∗∗∗∗*p* < 0.0001 by unpaired *t* test (*A*), one-way ANOVA with Tukey's post hoc test (*B* and *D*) or two-way ANOVA with Tukey's post hoc test (*E* and *F*). Cav-3, caveolin-3; CMKO, cardiomyocyte-specific RORα KO; IP, immunopulldown.

Analysis of Cav-3 protein with immunoblotting demonstrated that the level of Cav-3 was 34% lower in RORα^sg/sg^ than WT hearts (Fig. 6*A*). In the CMKO hearts, Cav-3 mRNA (Fig. 6*B*) and protein (Fig. 6, *C* and *D*) levels were similar to those in WT hearts in normoxic conditions. After WT and CMKO mice spent 8 h in a hypoxia chamber (10% O_2_), Cav-3 mRNA and protein were increased 2- to 3-fold in WT hearts, but there was no change in Cav-3 abundance in CMKO hearts (Fig. 6, *C* and *D*). These findings suggested that RORα may regulate Cav-3 expression in cardiomyocytes, particularly under hypoxic conditions.

Through promoter analysis, we found two consensus RORα-binding sites (ROR response elements [ROREs]) in the proximal promoter of the *Cav3* gene. To determine whether *Cav3* is a direct transcriptional target gene of RORα in adult mouse ventricular myocytes, we performed chromatin immunoprecipitation (ChIP)-PCR using immunopulldown with an anti-RORα antibody or an anti-IgG antibody as a negative control. We found significant enrichment of the regions containing the two ROREs (−0.1 kb, and −0.5 kb regions), but not in a region of the promoter that does not contain an RORE (−4.2 kb), suggesting that RORα binds *Cav3* directly in normoxic and hypoxic conditions (Fig. 6*E*). To expand upon this novel finding, we carried out a luciferase reporter assay using H9c2 ventricular myoblasts transfected with a luciferase reporter plasmid containing the *Cav3* promoter region (−350 bp from the transcriptional start site), a beta-galactosidase plasmid, and either empty vector or different amounts of mouse RORα expression plasmid. We found that RORα caused a dose-dependent activation of the *Cav3* promoter as indicated by an increase in luciferase activity (Fig. 6*F*), indicating that RORα regulates *Cav3* transcription in both normoxia and hypoxia.

Next, we examined the effect of RORα expression on Cav-3 expression in H9c2 cardiomyoblasts infected with a lentivirus containing doxycycline-inducible RORα4 (H9c2-Flag-RORα4-HA), the predominant RORα isoform in the heart (Fig. S4*A*). Doxycycline induced RORα4 in a dose-dependent manner (Fig. S4*B*) and RORα4 was localized largely to nuclei, as expected (Fig. S4*C*). To determine whether this increase in RORα expression correlated with enhanced mitophagy, we quantified colocalization of immunofluorescent LC3B (red) with mitochondria (green) and identified roughly 4 times more mitophagy events in the cells in which RORα was induced by doxycycline (Fig. S4*D*).

**Figure 7:**
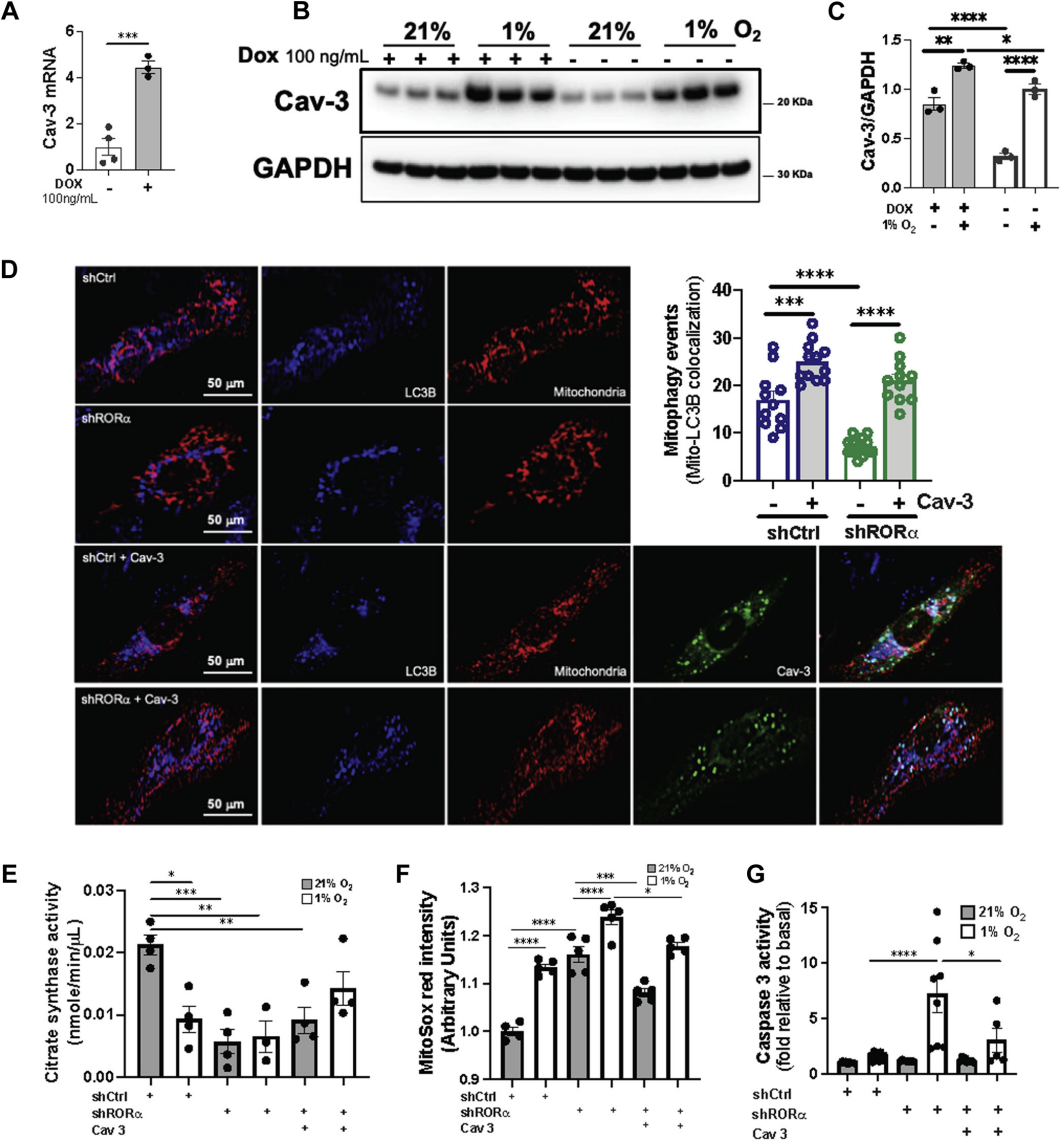
**Forced expression of caveolin-3 restores mitophagy and mitochondrial function after knockdown of RORα.***A*, quantitative RT-PCR for Cav-3 in H9c2-Flag/RORα4/HA cells. *B*, immunoblotting of cell lysates with (*C*) summary densitometry from H9c2-Flag/RORα4/HA cultured in normoxia (21% O_2_) or hypoxia (1% O_2_) for 24 h in the absence or presence of doxycycline (Dox) induction of RORα. *D*, H9c2-Flag/RORα4/HA cells with m-Raspberry-mito-7 with or without EGFP-Cav-3 plasmid transfection. *E*, citrate synthase assay for function mitochondrial mass. *F*, MitoSOX red for oxidative stress. *G*, caspase-3 activity assay for apoptosis. ∗*p* < 0.05, ∗∗*p* < 0.01, ∗∗∗*p* < 0.001, and ∗∗∗∗*p* < 0.0001 by unpaired *t* test (*A*) or one-way ANOVA with Tukey's post hoc test (*C*–*G*). Cav-3, caveolin-3; DAPI, 4′,6-diamidino-2-phenylindole; Dox, doxycycline; LC3, microtubule-associated protein 1A/1B-light chain 3.

The induction of RORα by doxycycline resulted in a 4.5-fold increase in Cav-3 expression (Fig. 7*A*). The expression of Cav-3 was then compared in doxycycline-treated and doxycycline-untreated H9c2-Flag-RORα4-HA cells exposed to either hypoxia (1% O_2_) or normoxia (21% O_2_). We found that hypoxia enhanced Cav-3 expression, and this upregulation was significantly greater in doxycycline-treated cells in which RORα was induced (Fig. 7, *B* and *C*). To determine whether Cav-3 is directly involved in the regulation of cardiomyocyte mitophagy by RORα, we knocked down RORα in H9c2 cardiomyoblasts with shRNA and then transfected these cells with an EGFP-Cav-3 plasmid. We then visualized mitophagy events by costaining for LC3B (blue) and mitochondria (red), using EGFP to identify Cav-3 (Fig. 7*D*). As expected, shRORα decreased mitophagy events. Forced expression of Cav-3 increased mitophagy events in both shCtrl and shRORα H9c2s and eliminated the mitophagy defect induced by RORα knockdown such that mitophagy events were equivalent in shCtrl-Cav-3+ and shRORα-Cav3+ H9c2s.

To determine the functional consequences of these findings, we assayed citrate synthase activity as a surrogate for functional mitochondrial mass, MitoSOX as an index of oxidative stress, caspase 3 activity as a marker of early apoptosis, and mitochondrial swelling as an indicator of mPTP opening. We found that reintroduction of Cav-3 abrogated shRORα-induced defects in citrate synthase activity (Fig. 7*E*) and mitigated shRORα-induced increases in oxidative stress (Fig. 7*F*), apoptosis (Fig. 7*G*), and mPTP opening (Fig. S5). These results collectively demonstrate that RORα-mediated Cav-3 transcription promotes mitophagy and preserves multiple critical aspects of mitochondrial function in normoxic and hypoxic cardiomyocytes. Taken together, these findings provide a novel mechanism for the mitochondria-protective effects of RORα in the heart.

## Discussion

In this study, we identified a novel role for the nuclear receptor RORα in promoting mitophagy through direct transcriptional upregulation of Cav-3. Loss of RORα profoundly compromised cardiomyocyte mitochondrial function leading to energy deprivation, oxidative stress, and cell death. These insults were particularly deleterious in the setting of hypoxia, a recognized inducer of mitophagy and a clinically relevant stressor. Nuclear receptors such as the peroxisome proliferator-activated receptor and estrogen-related receptor families are central regulators of cardiomyocyte mitochondrial structure and function (3), and the present findings identify RORα as another nuclear receptor that contributes to maintaining cardiac mitochondrial health. A role for RORα in regulating mitochondrial fission in the liver was proposed previously (25), but, to our knowledge, our present study is the first to demonstrate that RORα adaptively regulates mitophagy in any tissue.

We began these investigations to expand upon our earlier finding that the global absence of functional RORα in the RORα^sg/sg^ mouse model was associated with decreased mitochondrial number and ATP depletion in the heart (11). Here, we introduce a novel CMKO mouse model to help dissect cardiomyocyte autonomous from systemic effects of RORα loss of function. We found that both the RORα^sg/sg^ and CMKO mice exhibit compromised cardiac mitophagy in normoxic conditions and the absence of RORα from cardiomyocytes in the CMKO mouse markedly impairs induction of mitophagy. As mitophagy is a complex multistep process, we embarked on a series of experiments to discern which aspect of mitophagy was affected by the absence of RORα. We identified altered expression of multiple key mitophagy mediators in both RORα^sg/sg^ and CMKO mouse hearts, suggesting the possibility that RORα regulates mitophagy at multiple stages and through multiple mechanisms arising from the activities of RORα in the cardiomyocyte. We also show that knocking down RORα in primary cardiomyocytes reduces citrate synthase activity and respiratory capacity while compromising mitochondrial membrane potential and increasing oxidative stress, the first detailed characterization of how RORα affects mitochondrial function. These *in vitro* studies reinforce the findings from our two loss-of-function mouse models indicating that the *in vivo* effects of RORα on mitophagy and mitochondrial function arise from the expression of RORα in cardiomyocytes, rather than systemic effects. The striking basal phenotype of the CMKO mouse, with decreased contractile function and enhanced oxidative stress, further reinforces the critical physiological significance of cardiomyocyte RORα. Importantly, the present data are insufficient to ascribe the contractile deficit to mitophagy defects alone. In another related project, we actively are investigating whether RORα regulates the expression of key sarcomeric proteins, potentially providing another mechanism for the hypocontractile phenotype.

Our previous work demonstrated that RORα mitigated Ang II-induced cardiac hypertrophy through the regulation of cardiomyocyte *IL6* and STAT3 signaling (11). Here, we identify a novel role for RORα in promoting cardiomyocyte mitophagy at least in part through enhancing *Cav3* transcription. RORα binds two sites in the *Cav3* promoter to regulate Cav-3 expression directly (Fig. 6, *E* and *F*) in cardiomyocytes. Cav-3 is the predominant caveolin isoform in muscle cells and serves primarily as a scaffold for membrane trafficking and signaling within caveolae (26). However, Cav-3 also dissociates from caveolae to the cytosol in injured cardiomyocytes (27) and to the mitochondrial membrane where it enhances respiratory capacity and mitigates oxidative stress (28). Cardiac-specific overexpression of Cav-3 protects against hypertrophy induced by pressure loading (29) and ischemia-reperfusion injury (30). Cav-3 KO mice exhibit impaired cardiomyocyte mitochondrial function (28) and enhanced susceptibility to injury (31). Recognized mechanisms for these protective effects include preservation of t-tubular calcium flux (32) and prevention of hypoxia-induced apoptosis (33).

The importance of caveolins in regulating autophagy has been established in cardiomyoblasts and other cell types. Cav-3 is upregulated by simulated ischemia-reperfusion in HL-1 cardiomyoblasts; here, we demonstrate that RORα likely contributes to this regulation. Knockdown of Cav-3 in HL-1s compromises autophagy and mitochondrial function, contributing to enhanced cell death (23). Cav-1 interacts with beclin-1 to induce autophagolysosome formation and protect against cerebral oxidative stress and ischemic injury (34). Here, we show that Cav-3 abundance is lower in the hearts of RORα^sg/sg^ mice (Fig. 6, *A* and *B*) and cardiomyocyte-specific RORα deletion is associated with a marked failure of the expected hypoxic induction of Cav-3 (Fig. 6, *C* and *D*). Conversely, RORα overexpression in cardiomyocytes induces Cav-3 expression (Fig. 7, *A*–*C*). Forced expression of Cav-3 rescues defects in mitophagosome formation (Fig. 7*D*) and broadly mitigates insults to mitochondrial function induced by shRORα knockdown in normoxic and hypoxic cardiomyocytes (Fig. 7, *E*–*G*), consistent with published roles for Cav-3 (23). These *in vitro* findings demonstrate the importance of the cardiomyocyte RORα-Cav-3 axis in regulating mitophagy and mitochondrial physiology in cardiomyocytes, particularly in the context of hypoxia.

It is important to acknowledge that although both global and cardiomyocyte-specific loss of RORα is associated with defects in normoxic mitophagy, the basal abundance of Cav-3 is normal in CMKO mice, whereas it is decreased in RORα^sg/sg^ mice. These findings indicate that RORα is not an essential transcription factor for basal Cav-3 expression and raise the possibility that systemic factors influence the cardiac expression of Cav-3 in normoxic conditions. Importantly, they also suggest that cardiomyocyte RORα may influence mitophagy through pathways that do not involve Cav-3. Indeed, we cannot exclude the possibility that RORα also directly regulates the transcript abundance of other key mitophagy mediators, as there are ROREs in the promoter regions of PINK1, PRKN, Bnip3, and LC3B. Nevertheless, taken together, our present findings suggest that RORα might regulate Cav-3 as part of a coordinated and protective response to hypoxia in the heart that includes enhanced mitophagy.

We found that RORα was upregulated by hypoxia in cardiomyocytes (Fig. 5, *A* and *B*) and that RORα knockdown broadly compromised cardiomyocyte mitochondrial function under hypoxic conditions (Fig. 5, *E*–*K*). We also show that our novel CMKO mice exhibit a failure to induce hypoxic mitophagy *in vivo* (Fig. 4). Although the role of RORα in the cardiac response to hypoxia has not been studied previously, a complex interaction has been demonstrated in other tissues between RORα and hypoxia-inducible factor-1α (HIF-1α), the canonical regulator of cellular response to low oxygen tension. RORα regulates the transcription of HIF-1α in endothelial cells (35). Interestingly, hypoxia increases RORα transcription in a hepatoma cell line by binding to a hypoxia response element in the RORα promoter (36), indicating the potential for reciprocal regulation of RORα and HIF-1α. In future studies, we will explore whether RORα regulates other critical elements of the cardiomyocyte response to hypoxia.

Although our central conclusion is that RORα regulates mitophagy to preserve the cardiomyocyte mitochondrial pool, we also present evidence that RORα may be involved in other aspects of mitochondrial homeostasis. We show that the expression of several key regulators of mitochondrial biogenesis (PGC1α, Gabpa, and TFAM) was lower in RORα^sg/sg^ than WT hearts (Fig. S1*A*). PGC1α is more abundant in RORα^sg/sg^ liver cells (37), but less abundant in skeletal muscle cells lacking functional RORα (24), indicating that this regulation is tissue dependent. We also show that Opa1, a canonical regulator of mitochondrial fission, is reduced in RORα^sg/sg^ hearts (Fig. S1*B*), consistent with a previous report that RORα regulates mitochondrial dynamics in hepatocytes (25). As such, it is possible that induction of mitophagy alone does not account for the protective effects of RORα on mitochondrial function. Indeed, in future studies, we will explore potential roles for RORα in the regulation of mitochondrial biogenesis and dynamics in cardiomyocytes.

In summary, our experiments indicate that RORα protects cardiomyocyte mitochondrial function through regulation of mitophagy, in normoxia as well as in the critical context of hypoxia. These findings provide mechanistic underpinning for recent studies demonstrating an emerging cardioprotective role for RORα in the setting of ischemia-reperfusion, diabetic cardiomyopathy, and Ang II-induced heart failure. The RORα agonist SR1078 recently was shown to be well tolerated and efficacious in an animal model of autism (38) and our present study indicates that this agonist is active in normoxic and hypoxic cardiomyocytes (Fig. 2, *E* and *G*). Interestingly, maresin-1, an endogenous RORα ligand, was recently shown to promote physiological cardiomyocyte hypertrophy through IGF-1 (39). Taken together, these studies suggest that activation of RORα *in vivo* could represent a novel therapeutic approach to heart disease.

## Experimental procedures

### Experimental animals

Heterozygous RORα^sg/sg^ mice on a C57BL/6J background were purchased from Jackson Laboratory and maintained as previously described (11). Homozygous RORα^sg/sg^ mice, the products of heterozygous breeding, and WT littermates were used in all experiments at 12 to 16 weeks of age. Homozygous CMKO mice were generated by crossing floxed RORα mice with LoxP sites flanking exons 9 to 11 (Anton Jetten, NIEHS) with αMHC-Cre mice (Dale Abel, University of Iowa). Except as noted, all CMWT and CMKO animals were 4 to 5 months old in all experiments. All animal studies followed the NIH Guide for the Care and Use of Laboratory Animals, and animal protocols were approved by the NIEHS Animal Care and Use Committee and the University of North Carolina Institutional Animal Care and Use Committee. For euthanasia, animals were anesthetized with 4% isoflurane by continuous inhalation until the toe pinch reflex was absent and then underwent cervical dislocation.

### TEM

Cardiac sections were observed with a LEO EM910 TEM operating at 80 kV (LEO Electron Microscopy) and photographed with a Gatan Orius SC1000 CCD Digital Camera and Digital Micrograph 3.11.0 (Gatan). The mitochondrial cross-sectional area was measured using the Area:Perimeter function in the NIH ImageJ Segmentation plugin at magnifications of ×5000 to ×10,000. The global scale was set according to the image-specific scale generated by the Gatan camera output. Intact mitochondria were defined by circumferential membrane containment. The length and width were measured from the outer mitochondrial membrane to outer mitochondrial membrane along the longest visible axis in each perpendicular direction. An average of 2500 mitochondria were analyzed from 30 fields from multiple levels from three hearts per mouse cohort. The mitochondrial number was counted from 12 randomly selected TEM sections from WT mice (n = 2) and 13 randomly selected TEM sections from RORα^sg/sg^ mice (n = 2), using the ImageJ Particle Analysis plugin tool.

### Hypoxia chamber

Mice were housed in a hypoxia chamber (OxyCycler) with internal dimension (76 × 51 × 51 cm) sufficient to hold four cages with four mice per cage as previously described (40). The inflow rate (nitrogen mixed with oxygen or room air) was ∼3.1 ft^3^/h, and four holes (0.7-cm diameter) were opened at the bottom of each of the chamber’s three sides. Drierite (#21909-5000, Acros) and soda lime (#36596, Alfa Aesar) were placed at the bottom of the chamber in 12 × 10 × 5 cm trays (∼260 g and 200 g, respectively). CO_2_ was measured using a COZIR Wide-Range sensor and GasLab software (#CM-0123 CO2meter.com). The hypoxia chambers have internal fans to prevent gradients in composition of the atmospheres. CO_2_ was automatically recorded at 5-min intervals. FIO_2_ was lowered from 21% to 10% (hypoxia) in ∼3 h. Control (21% O_2_, normoxia) mice were maintained in an identical chamber. Hypoxia chambers were generously provided by Dr James Faber.

### BFA1 injection in mice

Six- to 8-week-old male mice were injected with BFA1 or vehicle (PBS) 2 h before sacrifice. BFA1 (Sigma-Aldrich) was dissolved in 100 mg/ml DMSO and diluted to 0.1 mg/ml with PBS at the time of use and injected at 1 mg/kg by i.p. injection.

### Histology and immunofluorescence microscopy

Mice were heparinized, and the heart was perfused with 10 ml PBS followed by 20 ml of 4% paraformaldehyde (PFA)/PBS through a 23G butterfly needle, and then excised and placed in 4% PFA/PBS for 24 h before transfer to 70% ethanol. For immunohistochemistry, the hearts were fixed overnight in 4% PFA/PBS, incubated in 30% sucrose/PBS, and then frozen in O.T.C. medium (Tissue-Tek). Frozen sections (10 microns), obtained with a Leica cryostat (Leica), were placed on glass slides, dried at room temperature (RT), and then incubated with primary antibodies. After washing, the sections were incubated for 3 h at RT with anti-mouse, anti-rabbit, anti-goat, or anti-rat Alexa Fluor-488–, Fluor-594–, or Fluor-647–conjugated secondary antibodies (1:1000, Life Technologies). Fluorescence was observed with a Zeiss LSM880 confocal microscope.

### Quantification of Masson Trichrome staining

Heart sections were stained with Masson Trichrome to detect fibrosis. The percentage of Masson Trichrome–positive tissue was measured in an automated fashion using Fiji software (NIH) using the “Color Deconvolution,” “Area,” “Integrated Intensity,” and “Limit to Threshold” plug-ins.

### qRT-PCR

The total mouse heart RNA was isolated with an RNeasy mini kit (QIAGEN) or RNAqueous Micro RNA isolation kit (Ambion) following the manufacturer's instructions. RNA was reverse-transcribed using a High-Capacity cDNA Archive Kit (Applied Biosystems). qRT-PCRs were carried out in triplicate in a LightCycler 480 System (Roche) using either probe/primer sets or SYBR Green I (see primer list). Relative quantitation of PCR products used the ΔΔCt method relative to two validated reference genes (*Tbp1* and *Polr2a*). All probes and primers were from Roche or Thermo Fisher.

### Immunoblotting

Whole-tissue or whole-cell lysates were produced in RIPA buffer supplemented with PhosSTOP (Roche Diagnostics Corporation) and protease inhibitor cocktail (Roche Diagnostics Corporation). Subsequently samples were incubated in 4 × LDS sample buffer with 2% β-mercaptoethanol, for 10 min at 70 °C. SDS-PAGE and immunoblotting were performed using the 4 to 12% NuPAGE gel system (Life Technologies). Membranes were blocked in 5% milk/TBS-Tween, incubated in the primary antibody overnight at 4 °C and then secondary horseradish peroxidase (HRP)-conjugated antibodies for 1 h at RT. Images were generated using Amersham ECL Select Western Blotting Detection Reagent (GE Healthcare life sciences) and the MultiDoc-It Imaging System (UVP gel imaging system).

### Antibodies

RORα (GTX100029, 1:1000), SQSTM1/p62 (GTX629890, 1:1000), PINK1 (GTX107851, 1:1000), LC3B (GTX127375, 1:1000), Ambra1 (GTX55507, 1:1000), and HIF1α (GTX628480) were from Genetex. VDAC1 (#4661, 1:000), HA (#3724, 1:1000), LC3B (#83506, 1:500), PDH (#3205, 1:1000), ANT2/SLC25A5 (#14671, 1:1000), and HSP60 (#12165, 1:1000) were from Cell Signaling Technology. GAPDH (MAB374 clone 6C5, 1: 10,000, Millipore), Cav-3 (sc-5310, 1:1000), Parkin (sc-32282, 1:1000), and Bnip3 (sc-56167, 1:1000) were from Santa Cruz Biotechnology. Polyclonal goat anti-rabbit IgG-HRP (A9169, 1:5000), polyclonal rabbit anti-mouse IgG-HRP (A9044, 1:5000), and polyclonal rabbit anti-goat IgG-HRP (A5420, 1:5000) were from Sigma-Aldrich.

### NRVM cultures, immunocytochemistry, and lentiviral infections

Female Sprague-Dawley rats and newborn litters were from Charles River Laboratories. Neonates were deeply anesthetized with 4% inhaled isoflurane and then euthanized by decapitation, and NRVMs were isolated as previously described (41). Experiments were carried out after 36 to 48 h of serum starvation in the presence of insulin, transferrin, and BrdU. NRVM hypoxia was induced by incubation in a 37 °C hypoxia chamber with 1% O_2_ after infection with lentivirus containing either shCtrl or shRNA specifically targeting rat RORα (iO51217 or iV051217, abm). shRORα target sequences are as follows:

TGTCATTACGTGTGAAGGCTGCAAGGGCT, ACCTACAACATCTCAGCCAATGGGCTGAC, GGACTGGACATCAATGGGATCAAACCCGA, and AGAGGTGATGTGGCAGTTGTGTGCTATCA.

### Immunofluorescence for phagolysosomes in H9c2 myoblasts

H9c2 rat ventricular myoblasts were treated with vehicle, RORα agonist, or RORα inverse agonist for 24 h and then incubated with LysoTracker for 15 min at 37 °C with 5% CO_2_. Cells were fixed with 4% PFA and stained with anti-LC3B (autophagy marker) and anti-Hsp60 (mitochondria marker). For each coverslip costaining anti-LC3B, anti-Hsp60, and LysoTracker, 12 random images were acquired utilizing a Zeiss 880 at 40× oil immersion. Each image was deconvolved using the regularized inverse filter method on Zeiss Zen Pro software. Images were further processed in ImageJ to segment and count puncta containing three positive colors. Results of three positive puncta counts were copied into an Excel sheet and normalized to the number of cells (according to the 4′,6-diamidino-2-phenylindole counts).

### Mitochondrial fractionation

Mitochondria were isolated from frozen heart tissue using a Mitochondria isolation kit (ab110168, Abcam). Tissue was briefly washed in the isolation buffer, dried with the Whatman filter paper, weighed, placed in a glass beaker, thoroughly minced, and then homogenized with a Dounce homogenizer. The homogenate was centrifuged at 1000*g* for 10 min at 4 °C. The supernatant was centrifuged at 12,000*g* for 15 min at 4 °C and then saved as crude cytosolic and nuclear fractions, and the pellet was centrifuged again at 12,000*g* for 15 min after resuspending in the isolation buffer and protease inhibitor for further mitochondrial purification. The homogenized pellets were resuspended in the isolation buffer with protease and phosphatase inhibitor cocktails (Roche). The protein concentration was measured by the BCA protein assay kit (Thermo Scientific).

### ATP bioluminescence assay

The ATP content was determined using a luciferin-luciferase ATP assay (Thermo Fisher Scientific; A22066) according to the manufacturer’s protocol. All reagents were placed in ice before the experiments were carried out. The ATP solution (100 nM) was prepared by mixing 50 μl of a stock solution (5 mM) with 950 μl of deionized water and buffer in a microcentrifuge tube. The mixture was then transferred to a 96-well microplate for measurement of ATP bioluminescence. In brief, 100 μl of the ATP solution was mixed with 100 μl of the luciferin-luciferase reagent (10 mg/ml; reconstituted with deionized water) in each well of the microplate. ATP bioluminescence was measured immediately using a microplate luminometer. The integration time of the luminometer was set at 1 s with normal gain.

### Mitochondrial membrane potential

Mitochondrial membrane potential in NRVMs was determined by JC-1 reduction. Cells were incubated with JC-1 (Abcam) according to the manufacturer's protocol. In brief, serum-starved NRVMs were incubated in the normoxia chamber with 21% O_2_ or in the hypoxia chamber with 1% after infection with shCtrl or shRORα. JC-1 (2 μM) was added to each well for 30 min. Cells were washed once with the medium and then analyzed by the plate reader (CLARIOstar, BMG LABTECH). JC-1 green fluorescence was excited at 488 nm, and emission was detected using a 530 ± 40 nm filter. JC-1 red fluorescence was excited at 488 nm, and emission was detected using a 613 ± 20 nm filter.

### SDH activity assay

SDH activity was measured using an SDH Activity Colorimetric Assay Kit (MBS2540432) purchased from MyBiosource Co. After correcting for the background, SDH activity in NRVM lysates was determined.

### LDH release assay

For measurement of cytotoxicity, NRVMs were seeded onto 96-well plates. After treatment, 50 μl of the culture medium from each well was collected and transferred to another 96-well culture plate. 50 μl of the LDH reagent (Thermo Fisher Scientific, C20300) was then added to each well, and the solution incubated at RT for 30 min. After the reaction was complete, absorbance at 490 nm was measured by an ELISA reader.

### Citrate synthase activity

Citrate synthase activity assay kit (MAK 193, MilliporeSigma) was used in NRVMs incubated at 21% or 1% O_2_ after infection with shCtrl or shRORα lentivirus.

### Mitochondrial ROS measurement

The production of superoxide by the mitochondria of NRVMs incubated at 21% or 1% O_2_ after infection with shCtrl- or shRORα lentivirus was measured using MitoSOX Red mitochondrial superoxide indicator (M36008, Thermo Fisher Scientific).

### OCR assay

The OCR was measured using an extracellular oxygen consumption assay kit (ab197243; Abcam) following the manufacturer’s protocol. NRVMs in a 96-well black plate were incubated at 21% or 1% O_2_ after infection with shCtrl or shRORα lentivirus in triplicates for each condition. A negative control was prepared with the reaction buffer without cells. All wells were covered with two drops of preheated mineral oil to block the back diffusion of oxygen. The OCR was calculated based on fluorescent signals (excitation of 380 nm/emission of 650 nm), measured in 1.5-min intervals for 1.5 h using a plate reader (CLARIOstar, BMG LABTECH). The experiment was repeated two times independently.

### Keima with the mitochondrial localization signal

Keima with the mitochondrial localization signal, a mitochondrially localized pH-indicator protein (42), was generously supplied by Junichi Sadoshima (Rutgers New Jersey Medical School). The method used to detect lysosomal delivery of Keima with the mitochondrial localization signal has been described (22). Briefly, NRVMs were infected with shCtrl or shRORα lentivirus for 24 h and then infected with mt-Keima virus for visualizing mitophagy (543 nm and 458 nm) under fluorescence microscopy. Relative fluorescence intensity (ratio of 543 nm/458 nm) was determined by ImageJ.

### Treatment with RORα inverse agonist (SR3335) and agonist (SR1078)

NRVMs were treated with vehicle or RORα inverse agonist, SR3335 20 μM (43) or agonist, SR1078 2 μM (38) for 6 h, and then incubated in a hypoxia chamber (1% O_2_) after pretreatment with bafilomycin A (100 nM) for 2 h.

### ChIP-PCR

To demonstrate transcriptional regulation of Cav-3 by RORα, adult cardiomyocytes isolated from 3- to 4-month-old mice were washed in PBS containing protease and phosphatase inhibitor cocktail (Thermo Scientific) and then fixed in 1% formaldehyde at 25 °C for 8 min for cross-linking and followed by quenching by 0.125 M glycine at 25 °C for 8 min as previously described (11). The amount of chromatin immunoprecipitated DNA relative to each input DNA was determined by quantitative PCR in triplicate with primers amplifying consensus RORE (AGGTCA) or a nontargeting sequence in the mouse Cav-3 promoter.

ChIP-PCR primers were as follows:

Caveolin-3-4.2k, forward 5′-GCCAAGAGGGATCAGCAATA-3′ and reverse 5′-CAAGGGAAGGCATTTGACAT-3′;

Caveolin-3-0.5k, forward 5′- CCCCATGGCCTTAGTTTGAT-3′ and reverse 5′-TTATTTGCGTGCAAGTGAGC-3′;

Caveolin-3-0.1k, forward 5′-CAGGGTGGGAAGACTCTTGA-3′ and reverse 5′-GTCCCAGCTCTGTCTCTTGC-3′.

### Generation of doxycycline-inducible H9c2 cell line and immunocytochemistry

An H9c2 cell line with doxycycline-inducible RORα4 was adapted from a published report (44). Briefly, RORα4 tagged with N-terminal FLAG and C-terminal HA (FLAG-RORα4-HA) was inserted into lentiviral vector pIND20, and viral soup was generated in HEK293T cells. H9c2 cells were infected with lentiviral pIND20-FLAG-RORα4-HA, and stable cells were selected by 500 μg/ml of G418. Induction of RORα4 was examined by Western blot using the HA antibody after treatment of doxycycline. For the Cav-3 rescue experiment, we infected H9c2s with shRORα or shCtrl for 48 h and then transfected with an EGFP-Cav-3 plasmid (Addgene plasmid #68396) or empty EGFP plasmid and an mRaspberry-Mito-7 plasmid (Addgene #55931) to localize mitochondria. For visualization of mitophagy, H9c2s were stained with LC3B and LysoTracker (Thermo Fisher Scientific, L7525).

### Luciferase reporter assay

H9c2 cells were grown in DMEM containing 10% fetal bovine serum and penicillin/streptomycin. Cells were transfected using Lipofectamine 2000 (Invitrogen) with 50 ng of luciferase reporter plasmid (pGL4.27) containing the Cav-3 promoter region (−350 bp from Exon+1), 50 ng of CMV-driven beta-galactosidase plasmid, and 100 ng of either CMV-driven mouse RORα or a control plasmid. Twenty-four hours after transfection, cells were lysed with Passive Lysis Buffer (Promega), and luciferase activity was measured with a Luciferase Assay System (Promega) and beta-galactosidase measured with a Luminescent Beta-Galactosidase Detection kit II (Takara) according to the manufacturer’s protocols. Transfections were performed in triplicate, with each experiment repeated at least 2 times.

### Cell death assays by TUNEL staining

For TUNEL, tissue slides were incubated with the TUNEL assay kit; BrdU-red (Abcam). In all cases, isotype-matched nonspecific IgG was used as negative control. Slides were visualized under confocal microscopy (Zeiss LSM880) when indicated. Five fields (at 40× magnification) per slide were randomly selected, and the percentage of positive cells was quantified using ImageJ (NIH) software.

### Mitochondrial ROS assays in isolated heart mitochondria and H9c2 cells

For detection of mitochondrial ROS formation, isolated mitochondria (0.5 mg/ml) were incubated with 5 mM H_2_DCFDA (Abcam) or 5 mM MitoSOX-Red (Thermo Fisher Scientific) for 30 min. Cells were infected with control or RORα-targeted shRNA. One hour before measurement, the cells were stained with 5 μM MitoSOX-Red. The fluorescence intensity was measured with a microplate reader at RT. The values at 525 nm (for H_2_DCFDA) or 580 nm (for MitoSOX-Red) were used to determine the fluorescence intensity.

### Caspase-3 activity assay

To examine cellular apoptosis, we used a caspase-3 colorimetric assay kit (K106-100, BioVision Inc). Enzyme reactions were performed in a 96-well microplate, and 5 μl of the cell lysate was added to each reaction mixture. Total protein quantification was performed in each sample by the Bradford assay using bovine serum albumin as the standard. Absorbance at 405 nm was measured using a microplate reader.

### Ca^2+^-induced mitochondrial swelling assay

Mitochondrial volume changes were monitored using a microplate reader at 540 nm. Measurements of swelling were carried out after adding 10 ml of 20 mM CaCl_2_ solution (500 nmol/mg mitochondrial protein) at 25 °C to isolated mitochondria (0.2 mg protein) from heart tissue or cardiomyocytes to trigger mitochondrial swelling. The incubation medium (125 mM KCl, 2 mM K_2_HPO_4_, 1 mM MgCl_2_, 20 mM Hepes, 5 mM glutamate, 5 mM malate, and 2 μM rotenone, pH 7.4) was supplemented with 50 μl of protease inhibitors stock solution (100× concentration). Measurements were made every 2 min for 30 min.

### Cloning Cav-3 promoter

The caveolin promoter (0.35 kb) was amplified using mouse genomic DNA with forward (5′-cagcaaacccctaatgtaagga-3′) and reverse primers (5′-ctcacgcgagaggagacct-3′) and cloned in pCR2.1. After confirming the sequence, the promoter was amplified with the forward (5′-ggtacccagcaaacccctaatgtaagga-3′) reverse primers (5′-aagcttctcacgcgagaggagacct-3′) and then cloned into pGL4.10 at KpnI and HindIII restriction sites. The sequence was verified at every step.

### Statistics

All results are presented as the mean ± SEM. Comparisons were made in GraphPad Prism 9 using unpaired *t* test (2 groups) or one-way ANOVA with Tukey’s post hoc analysis (>2 groups). A summary of numerical *p*-values and F-values for all data is available in Table S1.

## Data availability

All primary data are available upon request. All original Western blots are displayed in Figure S6.

## Supporting information

This article contains supporting information.

## Conflict of interest

The authors declare that they have no conflicts of interest with the contents of this article.
