# Supplementary material for: The nuclear receptor RORα preserves cardiomyocyte mitochondrial function by regulating caveolin-3-mediated mitophagy
Source: J Biol Chem. 2021 Oct 28;297(6):101358. doi: 10.1016/j.jbc.2021.101358 (PMC8626585; doi:10.1016/j.jbc.2021.101358)

Supporting Information, Figure S1

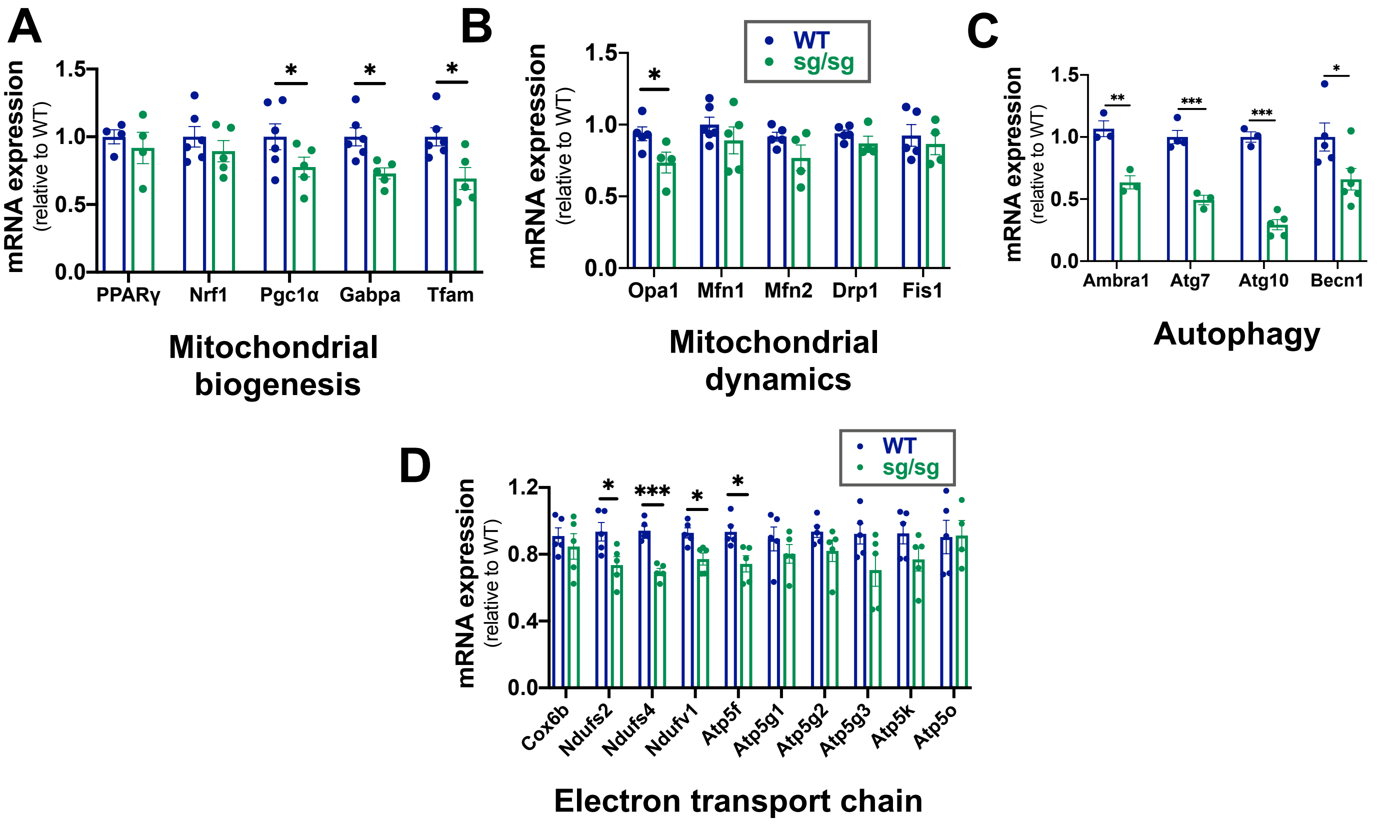

Figure S1. Quantitative reverse transcriptase PCR comparing abundance of selected transcripts involved in **(A)** mitochondrial biogenesis, **(B)** mitochondrial dynamics, **(C)** autophagy and **(D)** electron transport chain normalized to *Actb* and *Tbp* in wild type (WT) and ROR$\alpha^{sg/sg}$ (sg/sg) mice (n=6 per group).

Supporting Information, Figure S2

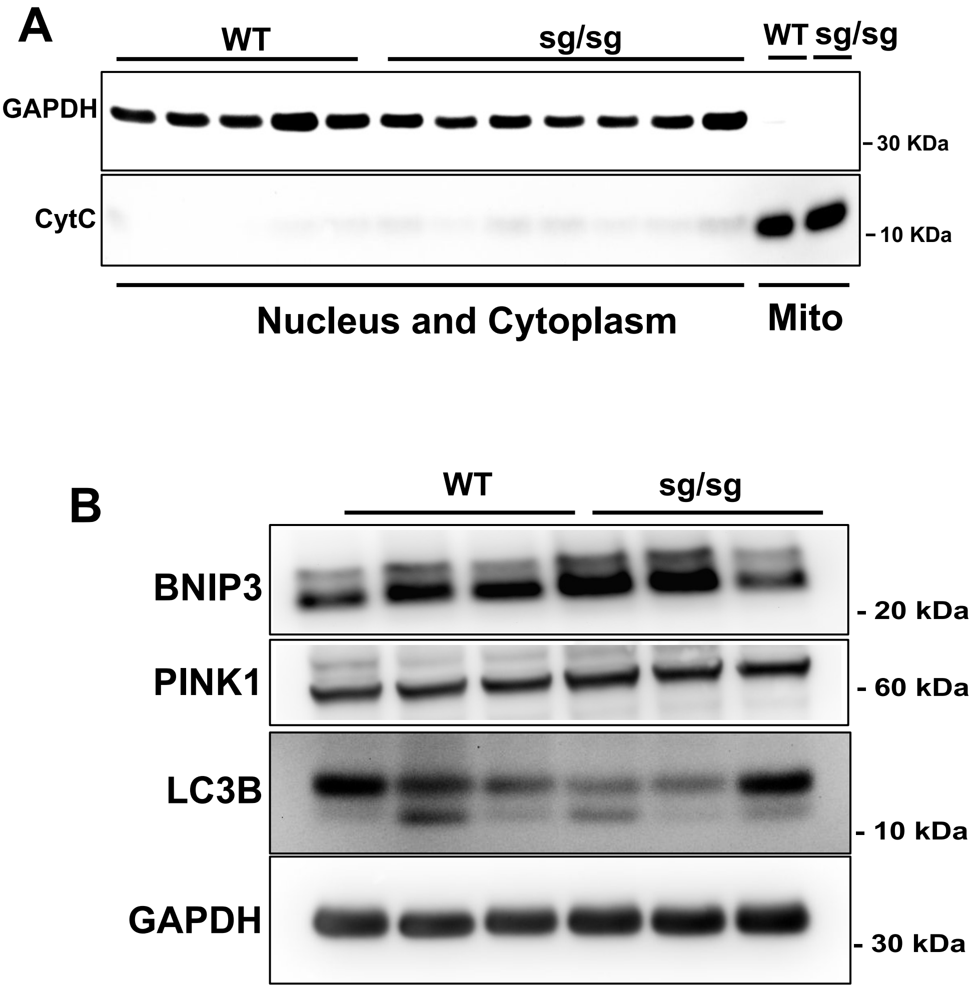

Figure S2. Immunoblotting of **(A)** fractionated and **(B)** whole heart lysates from wild type (WT) and staggerer (sg/sg) mice. Bnip3 = BCL2 interacting protein 3, Cyt C = cytochrome C oxidase; GAPDH = Glyceraldehyde 3-phosphate dehydrogenase, LC3B = microtubule-associated protein 1A/1B-light chain 3, Mito = mitochondrial; PINK1 = PTEN-induced kinase 1

Supporting Information, Figure S3

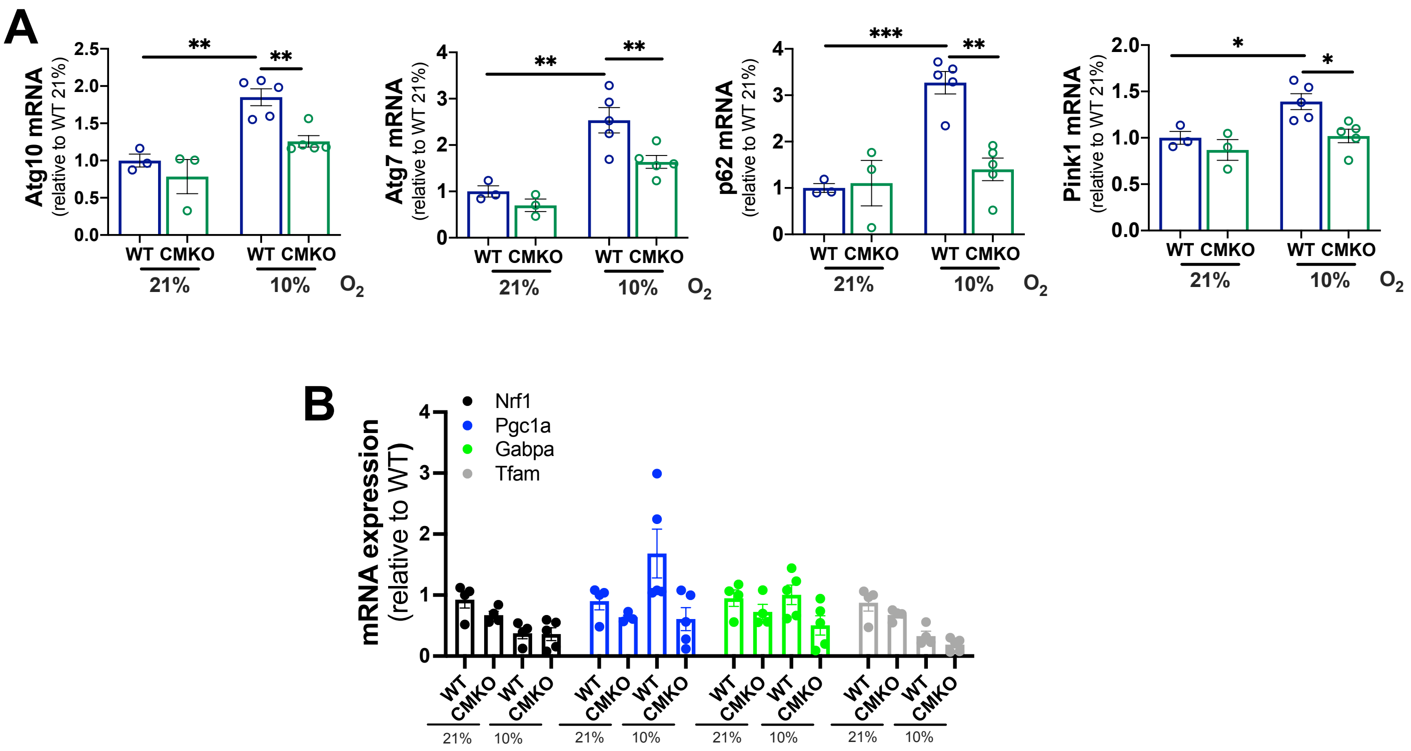

Figure S3. qRT-PCR for genes that induce **(A)** autophagy and **(B)** mitochondrial biogenesis in heart tissue from CMWT and CMKO mice exposed to normoxia (21% O_2) or to hypoxia (10% O_2) for 8 hrs.

Supporting Information, Figure S4

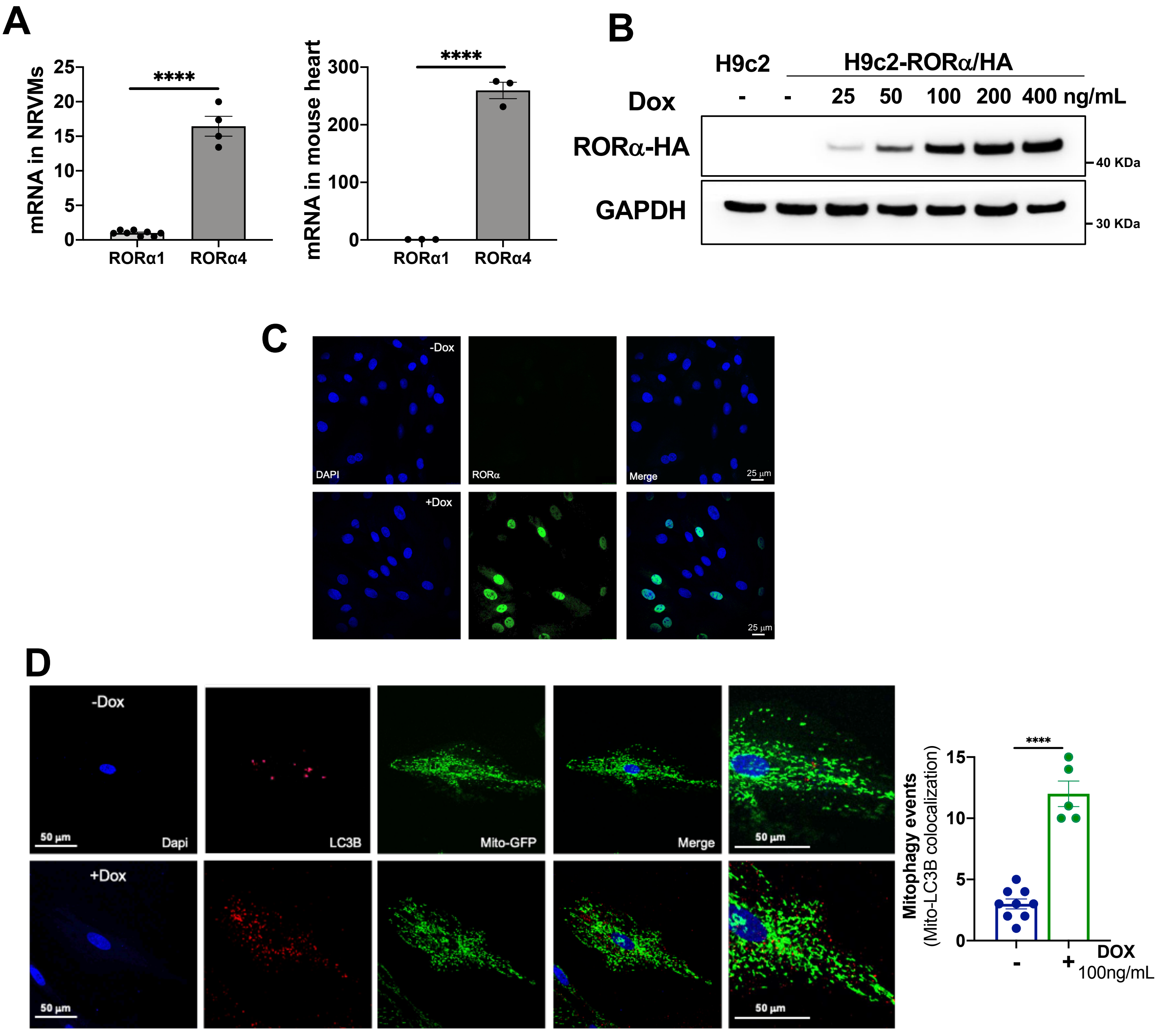

Figure S4. **(A)** Quantitative reverse transcriptase PCR for RORα subtypes in NRVMs and wild type mouse heart **(B)** Immunoblotting of cell lysates from H9c2 and H9c2-Flag/RORα4/HA with varying concentrations of doxycycline. **(C)** Immunofluorescence staining of HA antibody (RORα4, green) and DAPI (blue) in H9c2-Flag/RORα4/HA with or without doxycycline. **(D)** Immunofluorescence staining of H9c2-Flag/RORα4/HA cells with summary quantitation of LC3B and Mito-GFP co-staining (Image J);

Supporting Information, Figure S5

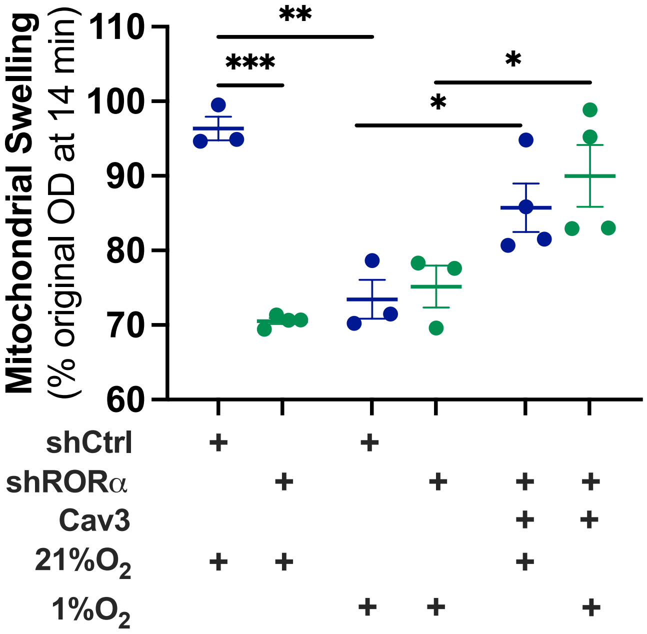

Supporting Figure S5. Mitochondrial swelling induced by calcium uptake and expressed as a percent of original optical density (OD). shCtrl = scrambled shRNA; shRORα = shRNA against RORα; Cav3 = lentivirius expressing caveolin-3.

Supporting Information, Figure S6

Figure 2A

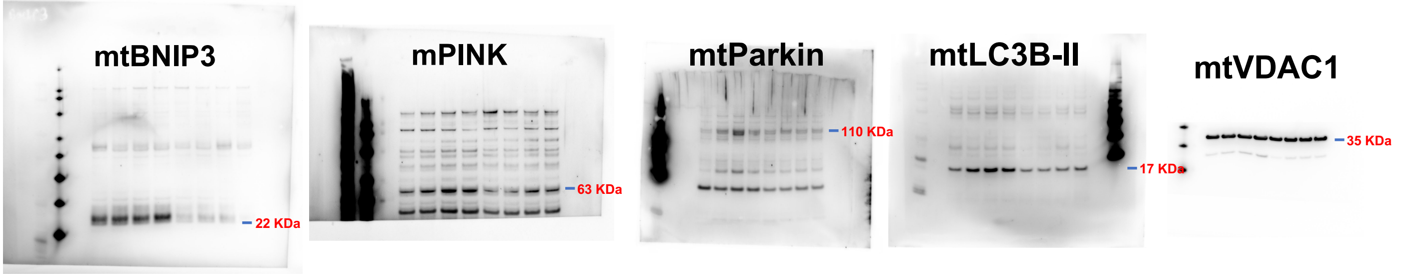

Figure 2C

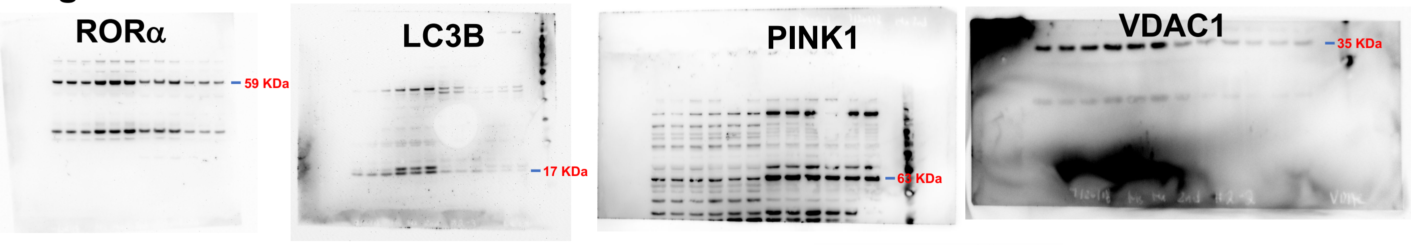

Figure 2F

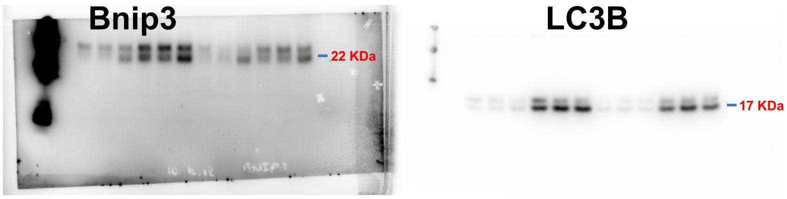

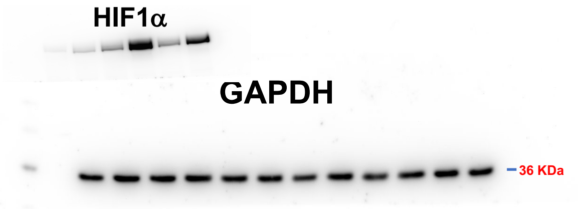

Figure 2G

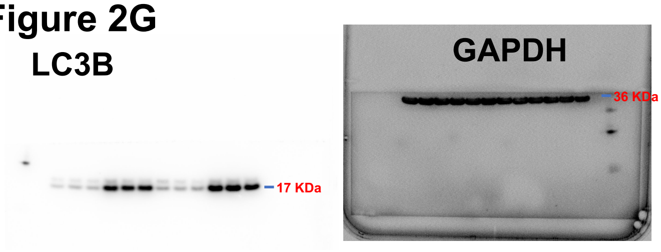

Figure 3C

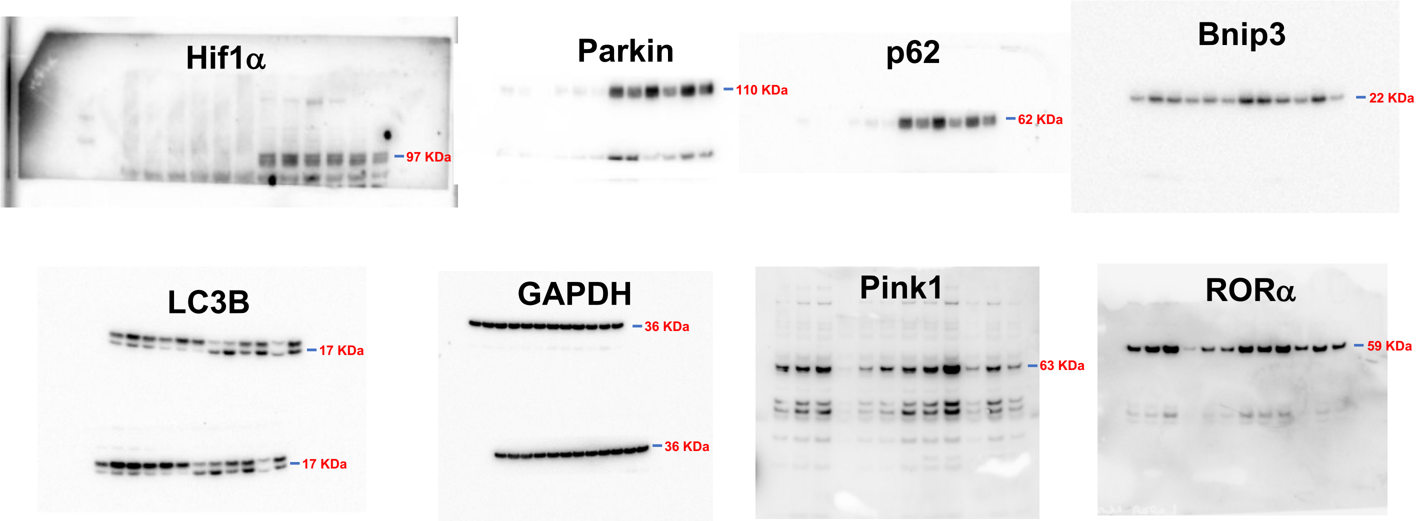

Figure S6. Original Western blots

Supporting Information, Figure S6 (cont)

Figure 4E

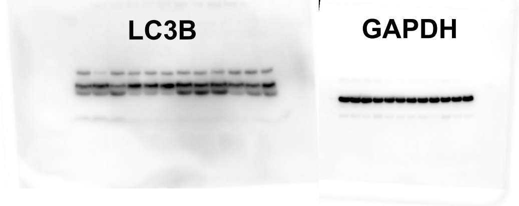

Figure 5B

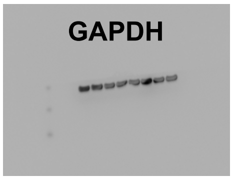

Figure 5C

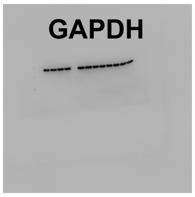

Figure 6C

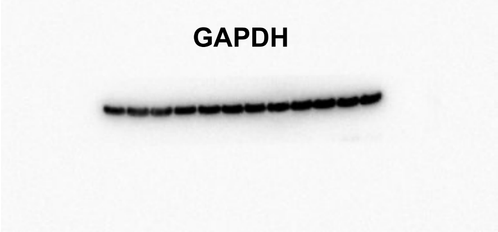

Supplement: Supplementary file 1 — Figures S1–S6 [file mmc1.pdf]
